# The Influence of the Exposome in the Cutaneous Squamous Cell Carcinoma, a Multicenter Case–Control Study

**DOI:** 10.3390/cancers15225376

**Published:** 2023-11-12

**Authors:** Alba Navarro-Bielsa, Tamara Gracia-Cazaña, Manuel Almagro, Sonia De la Fuente-Meira, Ángeles Flórez, Oriol Yélamos, Trinidad Montero-Vilchez, Carlos González-Cruz, Adrián Diago, Isabel Abadías-Granado, Victoria Fuentelsaz, María Colmenero, José Bañuls, Salvador Arias-Santiago, Agustín Buendía-Eisman, Manuel Almenara-Blasco, Pedro Gil-Pallares, Yolanda Gilaberte

**Affiliations:** 1 Department of Dermatology, Miguel Servet University Hospital, IIS Aragón, Universidad de Zaragoza, 50009 Zaragoza, Spain; 2 Department of Dermatology, Complejo Hospitalario Universitario A Coruña, 15006 A Coruña, Spain; 3 Department of Dermatology, Hospital Clínico Lozano Blesa, 50009 Zaragoza, Spain; 4 Department of Dermatology, University Hospital of Pontevedra, 36162 Pontevedra, Spain; 5 Department of Dermatology, Hospital de la Santa Creu i Sant Pau, IIB SANT PAU, Universitat Autònoma de Barcelona, 08193 Barcelona, Spain; 6 Department of Dermatology, Hospital Universitario Virgen de las Nieves, Instituto de Investigación IBS, 18012 Granada, Spain; 7 Department of Dermatology, Hospital Universitari Vall d’Hebron, 08035 Barcelona, Spain; 8 Department of Dermatology, Hospital de Barbastro, 22300 Huesca, Spain; 9 Department of Dermatology, Hospital Royo Villanova, 50015 Zaragoza, Spain; 10 Department of Dermatology, Hospital Costa del Sol, 29603 Marbella, Spain; 11 Department of Dermatology, Hospital General Universitario de Alicante, ISABIAL, 03010 Alicante, Spain; 12 Facultad de Medicina, Universidad de Granada, 18071 Granada, Spain; 13 Department of Dermatology, Complejo Hospitalario Universitario de Ferrol, Universidad de Santiago de Compostela, 15705 A Coruña, Spain

**Keywords:** exposome, squamous cell carcinoma, diet, environmental exposure

## Abstract

**Simple Summary:**

The influence of different exposome factors on squamous cell carcinoma has been studied in several articles, although generally including a limited number of factors, especially chronic sun exposure. We carried out a prospective multicenter case–control study of patients with a history of squamous cell carcinoma and a control group with no previous history of skin cancer, in which we compared most of the exposome variables, including sun exposure, photoprotection habits, diet, pollution, stress, and lifestyle. We found a significant association between squamous cell carcinoma and multiple exposome-related factors besides chronic sun exposure in the Spanish population. A better understanding of the actual impact of exposome in this condition could help design primary prevention strategies targeted at specific populations or risk behaviors.

**Abstract:**

Introduction: The concept of exposome refers to the total of harmful and beneficial environmental exposures that can help predict the organism’s biological responses over time. Ultraviolet radiation (UVR) from sun exposure has been recognized as the main etiological agent of skin cancer, and squamous cell carcinoma (SCC) is one most commonly associated with chronic exposure. However, in recent years, evidence suggests that lifestyle, environmental pollution, and contaminants in water and food can have an influence. Objectives: To study the relationship between SCC and sun exposure, pollution, stress, and lifestyle in a Spanish cohort. Materials and Method: A multicenter case–control study was carried out in which 13 dermatologists from different regions of Spain recruited cases and controls between April 2020 and August 2022. The group of cases were patients diagnosed with SCC and, as a control group, people who attended Dermatology consultations as companions with no history of skin cancer. Results: A total of 62 patients with SCC and 126 controls were included (62.9% males, median age 76.46 (10.1) and 33.3%, median age 55.7 (15), respectively). The SCC group had experienced more outside work than the controls (75% vs. 22.4%, *p* < 0.001), less recreational exposure (sunbathing, *p* = 0.05, and outdoor sports, *p* = 0.01), and a lower annual income (*p* = 0.01), with an increase in tobacco exposure (*p* < 0.001), without differences in other carcinogens, such as ionizing radiation or chemical exposure. The control group had a higher daily screentime use (*p* < 0.001) and practiced more relaxation activities (*p* = 0.03). A higher linolenic acid intake and lower coffee consumption were the only dietary variables associated with SCC (*p* < 0.05). Some chronic medications (anxiolytics, antidepressants, beta-blockers, statins, hydrochlorothiazide, ACE inhibitors, metformin, and omeprazole) were also statistically associated with SCC. Statistical significance for all aforementioned variables was maintained in the multivariate analysis (*p* < 0.05). Conclusions: The study found a significant association between SCC and multiple exposome-related factors in addition to chronic sun exposure in the Spanish population. Primary prevention strategies should target specific populations, such as outdoor workers promoting sun-safe behaviors and stress-reducing activities, in addition to adequate skin photoprotection in patients under certain medications associated with SCC.

## 1. Introduction

The concept of exposome refers to all the environmental exposure throughout human life and constitutes a new approach to the study of the role of the environment in human health [1]. Linked to this, the EXPOsOMICS project aims to assess environmental exposure, mainly environmental pollution and water contaminants, using “omic” techniques that can associate exposure data with biochemical and molecular changes. The results will help better understand how pollutants influence the risk of developing chronic diseases and different types of cancer [2]. Skin cancer is the most common cancer in humans. Its incidence has increased over the last 20 years, and in the next 20 years, an exponential increase close to 100% is predicted, leading to epidemic levels of prevalence [3]. In Spain, the crude incidence rates for squamous cell carcinoma (SCC) are 38.16 (CI 95%, 31.72–39.97) cases per 100,000 person-year [4]. 

As the skin is our most external organ and, therefore, it is more in contact with the environment, it is undoubtedly the most exposed organ to the action of everything that happens around us. Of all of them, ultraviolet radiation (UVR) from sunlight has been recognized as the main etiological agent for skin cancer development [5]. However, in recent years, there has been increasing evidence that environmental pollution and contaminants in water, food, or lifestyle can also have an influence. On the other hand, in a holistic concept of health and taking into account the interaction between the psyche, the nervous system, and the gut, besides the endocrine and immune systems, it is increasingly necessary to consider the influence of stress and sleep on the appearance of cancer [6].

The aim of this study was to describe and analyze the association between SCC and the exposome variables related to sun exposure, diet, pollution, stress, and lifestyle of the Spanish population.

## 2. Participants and Methods

### 2.1. Study Design

A multicenter case–control study was carried out by 13 dermatologists from different regions of Spain. The study was conducted between 1 April 2020 and 31 August 2022. The groups of cases included patients diagnosed with incidental SCC, histopathologically confirmed and control group of people who attended Dermatology offices as companions of these patients, of similar age and sex, with no personal history of skin cancer. The following inclusion criteria were applied: patients diagnosed with SCC (diagnosed a maximum of 3 months before the start of the study) and those that were diagnosed prospectively from the beginning of the inclusion period. The exclusion criteria were the following: age < 18 years; patients with photosensitive diseases; patients who did not sign the informed consent; patients with skin tumors diagnosed more than three months before the start of the study.

The variables collected were as follows: age, sex, marital status, income, height, weight, body mass index (BMI), place of residence (rural/urban), profession, phenotype and phototype, and chronic medication; sun exposure and photoprotection habits were evaluated using a validated questionnaire previously used by our group [7,8]; diet was evaluated using the validated PREDIMED questionnaire [9]; exposure to pollution, toxic substances, and ionizing radiation was reported by participants; stress was evaluated using the Perceived Stress Scale (PSS) [10,11]; number of hours of sport practice (outdoors and indoors) and number of hours of sleep was also recorded (Supplementary Material S1).

A Google Forms form was designed to collect all variables anonymously, and those results were later included in an Excel spreadsheet.

### 2.2. Statistical Analysis

Descriptive statistical analysis was performed for all variables. Continuous variables were presented as the number of valid cases, mean, standard deviation (SD), and 25th and 75th percentiles, depending on the results of the Kolmogorov–Smirnov test. Categorical variables were presented as the mean of the absolute and relative frequencies of each category over the total number of valid values (N).

Categorical variables were compared using the Chi-squared test. In the case of continuous variables, ANOVA was used. Logistic regression was used to determine which variables were associated with a diagnosis of SCC and for bias control. For all comparisons, statistical significance was set at *p* < 0.05.

Statistical analysis was performed using SAS (Statistical Analysis System) version 9.4 on the Windows platform.

### 2.3. Ethical Concerns

The present study was strictly observational, and the protocol was approved by the Aragón Ethical Committee for Clinical Research (C.I. PI19/311). All participants provided written informed consent prior to their enrolment.

## 3. Results

### 3.1. Demographic and Clinical Characteristics of the Sample/Study Population

The characteristics of the study population are presented in Table 1.

A total of 62 patients with SCC and 126 controls were included (62.9% males, median age 76.46 (10.1) and 33.3% males, median age 55.7 (15), respectively), without differences between anthropometric variables (weight, height, and BMI). Nevertheless, there were differences in hair and eye color and phototype; the SCC group had lighter eye (*p* = 0.003) and hair color (redhead, blond, and light brown) (*p* = 0.06) and a lower phototype grade (I to III) than the control group (*p* = 0.02).

The most frequent locations of SCC were the head and neck (72.6%), followed by the trunk (14.5%) and lower and upper extremities (11.3% and 8.1%). Among SCC patients, 45.1% had had a previous diagnosis of skin cancer (57.14% basal cell carcinoma, 42.85% SCC, and 3.57% melanoma). Regarding family history of skin cancer, there were no differences between cases and controls (14.8% vs. 22.7%). 

Most of the SCC and control groups were married (55% and 69.6%), but there were more single people in the control group (20.8% vs. 11.7%) and fewer widowed people (4% vs. 30%). There were differences in annual income; most SCC patients earned less than EUR 15,000/year (42.6% vs. 20.8% in controls), and there were no differences in residential environment (80.3% and 80.8% lived in urban environments). However, the groups differed in terms of workplaces: 28.2% of the SCC group worked outdoors at the moment of diagnosis, and 75% had previously worked outdoors, as compared with 5.2% and 22.4% in the control group; additionally, considering only the group of participants working outdoors, the number of daily hours spent outdoors was higher in the SCC patients than in the controls (7.2 (2.81) vs. 4.36 (2.66) h, *p* = 0.03), as was the number of years working outdoors (29.96 (17.52) vs. 15.45 (10.82), *p* = 0.01). 

Regarding exposure to other possible carcinogens like chemicals (pesticides, arsenic, coal tar, anthracenes, paraffins, asphalt, mineral oils, petroleum, and others) or ionizing radiation, there were no statistically significant differences between both groups.

### 3.2. Chronic Medication

Significant differences in medication were observed between the groups. A higher percentage of patients with SCC than controls took more anxiolytics (26.2% vs. 13%, *p* = 0.02), antidepressants (21.3% vs. 7%, *p* = 0.005), statins (41% vs. 14.8%, *p* < 0.001), beta-blockers (14.8% vs. 5.2%, *p* = 0.03), hydrochlorothiazide (14.8% vs. 1.7%, *p* < 0.001), angiotensin-converting enzyme (ACE) inhibitors (24.6% vs. 6.1%, *p* < 0.001), metformin (18% vs. 5.2%, *p* = 0.006), and omeprazole (39.3% vs. 16.5%, *p* < 0.001). Interestingly, no statistical differences between the intake of NSAIDS (including acetylsalicylic acid) or vitamin D were found. The characteristics of chronic medications in the sample are summarized in Table 2.

### 3.3. Sun Exposure Habits and Practices 

The characteristics of sun exposure and photoprotection measures are summarized in Table 3.

Regarding sun exposure habits, there were differences in recreational exposure (sunbathing, *p* = 0.05 or sport exposure, *p* = 0.004) between groups: 49.2% and 50.9% of the SCC group never did activities like sunbathing or outdoor sports, respectively, vs. control people, who 42.1% and 27.8% did sunbathing and outdoor sports between 6 and 30 days, although with similar days a year and hours a day of UVR exposure. 

Avoiding the hours of higher ultraviolet radiation between 12PM and 4PM was the most commonly used photoprotection measure by all participants, 67.2% and 62.6% always or habitually, followed by staying in the shade in the SCC group and sunscreen (SPF ≥ 30) in the controls, which was used always or habitually by 54.4% and 55.6%, respectively, and there were statistically significant differences between the SCC and control groups for this last one because the SCC group used less sunscreen than the controls (*p* = 0.05). 

The use of sunglasses (44.1% and 52.8% always or habitually) was similar for cases and controls, but the use of a hat or cap (44.1% in the SCC group vs. 20.8% in the controls always or habitually) obtained differences (*p* = 0.03). Finally, the use of clothes was the measure least used without differences between groups (28.2% and 26% always or habitually).

When the participants were asked if 15 years ago they were more exposed to ultraviolet radiation, the majority answered “yes”, although it was significantly higher in the group of SCC (79.3% vs. 62.9% controls, *p* = 0.02). When asked about the SPF of sunscreen used 15 years ago, there were differences between groups (*p* < 0.001); most of the controls used SPF > 21 (30.6% vs. 17.3%) and > 50 (28.1% vs. 9.6%) compared with the SCC group; however, this paradigm changed when they were asked what SPF they currently used, since cases and controls were using at least an SPF of 21 to 50 and the majority used an SPF > 50 (66.7% cases and 50% controls).

### 3.4. Diet

The dietary intake of 59 nutrients was calculated using the PREDIMED questionnaire (Supplementary Material, Table S1). Linolenic acid was the only nutrient statistically associated with SCC. Patients with SCC had a higher linolenic acid intake vs. controls (1.89 vs. 1.40, mcg/day *p* = 0.04). On the other hand, caffeinated coffee intake was higher in controls than in SCC patients (3.55 vs. 2.5 coffees per day, *p* = 0.01). No associations were found with the rest of the dietary variables analyzed.

### 3.5. Lifestyle and Stress

The characteristics of lifestyle and stress are presented in Table 4.

The controls did more relaxation exercises, meditation, mindfulness, or yoga (23.2% vs. 6.7%, *p* = 0.006) as well as practiced more sport (65% vs. 45.8%, *p* = 0.006), but without statistical differences between outdoor or indoor sport. The controls spent more hours using screens (50% >3 h vs. 12.7% *p* < 0.001), with no differences in perceived stress. Otherwise, the SCC group slept more hours a day than the controls (39% more than 8 h and 20.3% more than 10 h vs. 26.4% and 0.8%, respectively).

There were differences regarding smoking habits (*p* = 0.002); more SSC cases smoked in the past (44.1% vs. 20.2%), although now there were more smokers among the controls (21.9% vs. 10.2%).

Finally, differences were found in sunburns; almost 30% of the SCC group had two or more sunburns last year (*p* < 0.001).

### 3.6. Multivariate Analysis

**Figure 1:**
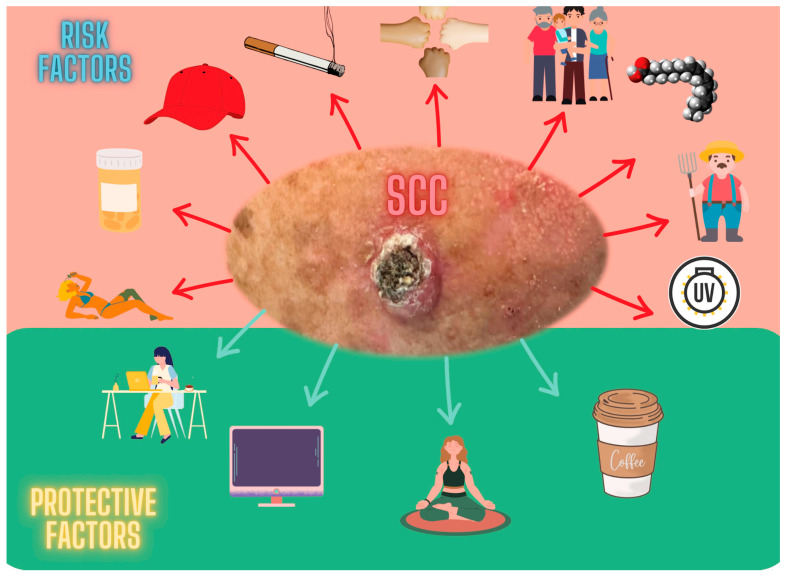
Exposome factors related to squamous cell carcinoma (SCC). In red, at the top, from left to right, risk factors for the development of SCC: exposition to ultraviolet radiation, drug consumption (anxiolytics, antidepressants, beta-blockers, statins, hydrochlorothiazide, ACE inhibitors, metformin, and omeprazole), use of hat or cap, smoking, phototype, age, linolenic acid intake, previous outside work, and years of sun exposure. At the bottom, in green, protective factors for the development of SCC from left to right: current work place indoors, hours/day with screens, relaxation activities, and coffee intake.

All the variables were included in a multivariate analysis to estimate the strength of the association between each variable and eliminate any confounding factors (Table 5 and Figure 1).

Genetic variables like hair color and phototype maintained their association in the multivariate analysis, as well as variables related to age and sun exposure (workplace, hours, and years of exposure) and photoprotection (the use of a hat and/or cap), the latest being a protective factor. Practicing relaxation activities and spending time in front of screens maintained their association as protective factors. Finally, chronic exposure to carcinogens such as tobacco and treatment with drugs such as antidepressants, beta-blockers, statins, hydrochlorothiazide, ACE inhibitors, metformin, and omeprazole continued to be associated with the development of SCC, with hydrochlorothiazide having the highest coefficient. Regarding diet, linolenic acid intake seems to be a risk factor, whereas caffeinated coffee intake was a protective factor.

### 3.7. Discussion

The present analysis of the exposome of SCC highlights the relevance of variables related to sun exposure: professional and chronic sun exposure more than recreational, and sun protection habits used in the past (at least 15 years ago) more than current measures. This confirms the relevance of sunlight in the development of this tumor, especially in relation to lighter skin phenotypes. The fact that the use of screens and relaxing activities, both usually associated with indoor activities, were more frequent in controls supports the importance of outdoor exposure in the development of SCC. Systemic factors, such as smoking or exposure to different types of drugs, especially hydrochlorothiazide, and a few nutrients, such as linolenic acid and coffee consumption, also seem to contribute to the development of this type of skin cancer. 

The characteristics of SCC are superimposable to those described in the literature, which appear to be more common in people of an older age with Fitzpatrick skin types I and II and are associated with lighter eye and hair colors [12]. In locations more exposed to UVR, epidemiologic studies indicate that cumulative sun exposure (principally ultraviolet B (UVB) radiation) is the most important environmental cause of SCC, the locations being the face, scalp, and neck for men and the face and legs for women [13]. Furthermore, individuals with a history of SCC are at increased risk for subsequent lesions [14]. 

A study among patients with at least two keratinocyte carcinomas reported that patients who had a previous SCC in situ had a 2-fold higher risk of developing additional invasive SCC [15]. 

We found differences in economic status; the SCC group had a lower income than the controls, which would be consistent with less recreational or leisure sun exposure, and differences between marital status in cases and controls are probably due to the difference in age, since controls were younger than the cases. However, other authors have shown that the difference in incomes leads to differences in the form and presentation of skin cancer. In Germany, a study was carried out on 70 million inhabitants, finding a direct correlation between having higher incomes and a better educational level with a higher prevalence of melanoma and non-melanoma skin cancer [16], and a multicenter study in five European countries (France, Germany, Portugal, Italy, and Sweden) found that there is a certain increased risk of skin cancer associated with a higher socioeconomic level in middle-aged patients, with no differences found in older patients [17].

Although most of our population lived in an urban environment, the SCC group worked more outdoors and for longer than the controls. It is known that UVR is recognized as the main etiological agent of skin cancer [5] and in recent years, there has been an increasing interest in this occupational UVR exposure, with many articles highlighting a higher risk of non-melanoma skin cancer development in outdoor workers, such as mountain guides, farm workers, or ski resort workers [8,18]. SCC is the skin cancer most attributed to accumulative and occupational UVR, whereas basal cell carcinoma and melanoma are attributed to sporadic and intense exposure (sunburns and childhood exposure). Our study supports these data but also found more sunburns in the cases than in controls [19].

We did not find any association between SCC and exposure to ionizing radiation or pesticides, even though the first one has been established as a cause of non-melanoma skin cancer [20]. 

We did not find any difference between living in a rural or urban environment, which could be related to pollution. In fact, some studies on animals and cultured cells support the synergic carcinogenic role between UVA radiation and Benzo[a]pyrene with SCC [21,22].

It is important to take into account that ionizing radiation and chemical exposure could have been underestimated in our sample, as well as in the general population, because they are not visible or perceptible, and people could be exposed to them many times without being aware of it.

Regarding drugs, a large number of medications have been implicated in the literature as causes of photosensitivity, which could be the major cause of the relationship between drugs and SCC [23]. 

Although in our study we have not identified a relationship between NSAIDs and SCC, there are other studies in which NSAIDs have been associated with a reduced risk of SCC (HR 0.77, 95% CI 0.64–0.93) [24], and pharmacologic agents that inhibit the enzyme COX-2 may be effective chemo-preventive agents for NMSC [25].

Anxiolytics have been associated with SCC development in our study in contrast with other studies in which midazolam inhibits the proliferation of SCC by downregulating p300 (resulting in increased expression of p21 and p27 and decreased expression of p-Rb) [26]. Similarly, antidepressants such as desipramine increase the expression of the p21 and p27 genes, inducing both apoptosis in SCC [27], but on the other hand, molecules such as N-nitrosomethylbenzylamine (NMBA), contained in some antidepressants, have shown a carcinogenicity effect via more than one pathway, which may act together to produce combination effects in the development of esophageal SCC [28].

Beta-blockers such as propranolol may be effective in SCC in vitro, downregulating p-P65 NF-ĸB and VEGF expression and cell migration [29]. In fact, β-adrenergic receptors increase tumorigenesis, stimulate cell proliferation, and inhibit apoptosis, and preclinical studies have also shown that β-adrenergic blockade can decrease the tumor burden [30].

The studies about statins are contradictory. A meta-analysis found no significant association between statin use and non-melanoma skin cancer [31]. Preclinical studies have suggested that statins could be considered chemo-preventive agents against cancer because of their antiproliferative, proapoptotic, antimetastatic, and anti-inflammatory properties [32]. However, the photosensitizing and immune-modulating effects of statins could increase the risk of skin cancer [33,34].

Thiazides and thiazide-like diuretics and ACE inhibitors have been associated with an increased risk of non-melanoma skin cancers [35,36]. Studies have suggested that antihypertensive drugs may increase the risk of these tumors, particularly hydrochlorothiazide, due to its photosensitizing properties [37], which is clearly associated with high cumulative hydrochlorothiazide use and the risk of SCC (adjusted hazard ratio 19.63, 95% CI 3.12–123.56). This is consistent with our findings, which show, in fact, the highest coefficient for this drug of all medications [38].

There is a study that demonstrates metformin-mediated immune antitumorigenic function through NK cell-mediated cytotoxicity and the downregulation of CXCL1 in SCC [39]. Although a recent meta-analysis based on the current evidence shows no significant association between metformin and the risk of skin cancer [40]. Finally, no study relates omeprazole use to the risk of SCC; however, omeprazole is included among photosensitizing drugs, which could explain this relationship [33,41].

We observed differences in photoprotection measures between groups, especially the increased use of hats in the SCC group. While most head coverings protect the scalp and forehead, many fail to cover the rest of the face and neck; large brimmed hats provide greater facial protection, except around midday [42]. Hats are important for photoprotection, but if the brim is not enough and the use of sunscreen is low, as observed in our cohort with SCC, the photoprotection may be insufficient. In response to questions on photoprotectors used in the previous 15 years, all participants reported that they used sunscreen with a lower SPF less frequently compared with the present, perhaps due to poorer knowledge of sun damage and the implications thereof. In recent years, campaigns promoting photoprotection measures have increased exponentially, increasing awareness among the general public.

Regarding dietary intakes, only linolenic acid and caffeinated coffee were associated with SCC among more than 59 items analyzed. The levels of linolenic acid were associated with a higher SCC risk, and this would be explained by some studies in animals where tumor-promoting effects of omega-6 fatty acids on UVR-induced carcinogenesis were found. These studies showed that the concentration of omega-6 fat intake proportionally increased with prostaglandin E synthase type 2 levels, which has a pro-inflammatory and immunosuppressive action and has been associated with aggressive growth patterns of NMSC [43,44]. Although biological processes link polyunsaturated fatty acid levels (PUFAs) to cancer protection, observational studies examining the relationship between PUFAs and basal cell carcinomas and SCC have reported contrary findings. Therefore, cohort studies with a larger number of patients and longer exposure times are required [45].

Concerning coffee consumption, our findings are consistent with other studies. Oh et al. [46] concluded that, compared with those who drank coffee less than once a week, those who drank three or more cups per day had a lower risk of squamous cell carcinoma (HR, 0.33; 95% CI, 0.13–0.84). It has been suggested that inhibition of NLRP3 inflammasome activity by caffeine might be a potential mechanism to reduce the risk of SCC [47].

Regarding the relationship between sport practice and skin cancer, which is relevant in our sample, many studies have revealed that athletes who perform sports outdoors received much more UVR and therefore had a higher risk of skin cancer, especially mountain sports, due to snow reflectance [48]. Extensive epidemiological studies have shown that recreational activities such as sunbathing on the beach or water sport practice are associated with an increased risk of basal cell carcinoma, while skiing has been associated with an increased risk of SCC [49,50]. On the other hand, the controls practiced more sports than cases. Experimental studies have demonstrated that exercising reduces the risk of developing skin cancer, in part due to a higher secretion of circulating insulin-like growth factor (IGF-1) and an activation of p53 and p21, IGFBP-3, and PTEN [51].

Regarding perceived stress, chronic stress can increase the susceptibility to skin cancer by suppressing Type 1 cytokines and protective T cells while increasing the regulatory or suppressor T cell number and/or function [52].

In our sample, there were no differences in the perceived stress evaluated with the PSS scale, but differences were found in the relaxation activities, where the control group practiced more of them. Controls spent more hours in front of a screen, which could be explained by the fact that they were younger than the SCC group and they had more indoor workplaces. This could also explain the difference in hours of sleep because the SCC group slept much more than the controls.

Finally, regarding smoking habits, our study showed an increased risk of SCC in smokers; in experimental animal studies, it has been shown that exposure to UVR and cigarette smoke has a synergistic effect on the induction of SCC in mice [53], and a 2012 systematic review and meta-analysis of 25 cohort and case–control studies found that smoking was associated with a 50 percent increased risk of developing SCC (risk ratio (RR) 1.52, 95% CI 1.15–2.01) [54]. The etiopathogenesis is explained by tobacco smoke increasing epidermal proliferation and inducing ROS, which oxidize the fibroblast DNA. On the other hand, affected fibroblasts induce the secretion of IL1, IL-6, IL-8, fibroblast growth factor-basic (FGF), monocyte chemoattractant protein (MCP-1), and insulin-like growth factor 4 (IGFBP4), which stimulate the proliferation of keratinocytes, which favors carcinogenesis in the skin through its regulation of cell proliferation. Acute oxidative stress compromises DNA operating systems, lengthening repair intervals and increasing DNA damage in smokers [55,56].

Our study has some limitations, mostly related to the sample selection. One limitation of the present study is its sample size, determined in part by the complexity of the dietary questionnaire, which was too high for elderly patients diagnosed with SCC, which may have resulted in inadequate statistical power to detect differences in many exposome variables. The fact that control participants were selected from individuals accompanying SCC patients to medical consultations may have also introduced another bias, given that some may share common exposures with the SCC group. Furthermore, the mean age of the control group was 20 years younger than that of the SCC group, and this could also have introduced some bias in the results. Finally, the study was only carried out in one country.

The primary strength of our study is that, to our knowledge, it is the first to simultaneously evaluate the association between SCC and most of all the possible exposome factors.

## 4. Conclusions

Our analysis of the exposome in SCC patients confirms sun exposure, specifically chronic occupational exposure, as the exposome variable most strongly associated with SCC, especially in people with light hair and a phototype who are genetically predisposed. Insufficient photoprotection at younger ages may be an important risk factor, as those effects are unlikely to be modified by improved photoprotection habits later in life. Chronic smokers and the consumption of photosensitizing drugs, especially hydrochlorothiazide, should also be considered relevant risk factors for SCC, and patients on these regimens should be targeted by awareness campaigns emphasizing the importance of adequate photoprotection. The consumption of caffeinated coffee could provide beneficial effects in the fight against SCC. A good balance between indoor and outdoor activities, including screentime, relaxation activities, and sports, is important to reduce SCC incidence. Given the possibility that climate change may increase time spent outdoors as well as the levels of radiation to which outdoor workers are exposed, campaigns targeting this specific group, as well as the broader population, are needed to promote safe behaviors under the sun and to instill healthy photoprotective habits beginning in childhood.

## Figures and Tables

**Table 1:** Demographic characteristics of the study population.

Variable		SCC	Control	*p*-Value
Sex, *N* (%)	Male	39 (62.9%)	42 (33.3%)	<0.001
	Female	23 (37.1%)	84 (66.7%)	
Age, *Mean* (*SD*) [*P25*;*P75*]		76.46 (10.11) [71.0; 84.0]	55.77 (15.00) [45.5; 67.0]	<0.001
Height (cm), *Mean* (*SD*) [*P25*;*P75*]		166.03 (8.14) [160.0; 150.0]	165.73 (8.86) [160.0; 172.0]	0.826
Weight (kg), *Mean* (*SD*) [*P25*;*P75*]		74.07 (14.90) [68.0; 80.0]	70.51 (15.13) [59.0; 81.0]	0.137
BMI (kg/m^2^), *Mean* (*SD*) [*P25*;*P75*]		26.78 (4.36) [24.2; 27.9]	25.56 (4.57) [22.2; 28.2]	0.087
Hair Color, *N* (%)	Red	3 (5.1%)	-	0.003
	Blond	16 (27.1%)	13 (10.7%)	
	Light brown	14 (23.7%)	42 (34.4%)	
	Dark brown	20 (33.9%)	49 (40.2%)	
	Black	6 (10.2%)	18 (14.8%)	
Eye Color, *N* (%)	Blue	12 (20.3%)	13 (10.9%)	0.064
	Green	7 (11.9%)	15 (12.6%)	
	Dark green/brown	8 (13.6%)	13 (10.9%)	
	Light brown	19 (32.2%)	27 (22.7%)	
	Dark brown	13 (22.0%)	51 (42.9%)	
Phototype, *N* (%)	I	8 (13.6%)	3 (2.4%)	0.028
	II	19 (32.2%)	33 (26.6%)	
	III	21 (35.6%)	52 (41.9%)	
	IV	6 (10.2%)	21 (16.9%)	
	V	5 (8.5%)	15 (12.1%)	
Tumor location, *N* (%)	Head and neck	45 (72.6%)	-	-
	Trunk	9 (14.5%)	-	-
	Upper extremities	5 (8.1%)	-	-
	Lower extremities	7 (11.3%)	-	-
Personal history of skin cancer, *N* (%)	Yes	28 (45.1%)	-	-
Type of skin cancer, *N* (%)	Basal cell carcinoma	16 (57.14%)	-	-
	SCC	12 (42.85%)	-	-
	Melanoma	1 (3.57%)	-	-
Family history of skin cancer, *N* (%)	Yes	9 (14.8%)	27 (22.7%)	0.420
	No	41 (67.2%)	70 (58.8%)	
	Unknown	11 (18.0%)	22 (18.5%)	
Marital status, *N* (%)	Single	7 (11.7%)	26 (20.8%)	<0.001
	Married	33 (55.0%)	87 (69.6%)	
	Divorced	2 (3.3%)	7 (5.6%)	
	Widower	18 (30.0%)	5 (4.0%)	
Annual income, *N* (%)	<EUR 15,000/year	20 (42.6%)	21 (20.8%)	0.011
	EUR 15,000–25,000/year	19 (40.4%)	39 (38.6%)	
	EUR 25,000–50,000/year	6 (12.8%)	34 (33.7%)	
	>EUR 50,000/year	2 (4.3%)	7 (6.9%)	
Residential environment, *N* (%)	Urban	49 (80.3%)	101 (80.8%)	0.939
	Rural	12 (19.7%)	24 (19.2%)	
Current workplace, *N* (%)	Indoors	28 (71.8%)	110 (94.8%)	<0.001
	Outdoors	11 (28.2%)	6 (5.2%)	
Previously worked outdoors, *N* (%)	Yes	24 (75.0%)	11 (22.4%)	<0.001
	No	8 (25.0%)	38 (77.6%)	
Daily hours of occupational exposure, *Mean* (*SD*) [*P25*;*P75*]		7.2 (2.81) [5.0; 9.0]	4.36 (2.66) [2.0; 7.0]	0.036
Years of exposure, *Mean* (*SD*) [*P25*;*P75*]		29.96 (17.52) [20.0; 40.0]	15.45 (10.82) [5.0; 20.0]	0.016
Exposure to chemicals, *N* (%)	Yes	6 (10.7%)	13 (10.4%)	0.918
	No	46 (82.1%)	105 (84.0%)	
Exposure to ionizing radiation, *N* (%)	Yes	-	8 (6.5%)	0.119
	No	54 (93.1%)	105 (84.7%)	

Abbreviations: BMI, body mass index; N, number of subjects; P25, 25th percentile; P75, 75th percentile; SD, standard deviation.

**Table 2:** Chronic medications taken by study population.

Variable	SCC	Control	*p*-Value
Acetylsalicylic acid	3 (4.9%)	1 (0.9%)	0.086
NSAIDs	4 (6.6%)	10 (8.7%)	0.617
Anxiolytics	16 (26.2%)	15 (13.0%)	0.028
Antidepressants or hypnotics	13 (21.3%)	8 (7.0%)	0.005
Contraceptives	-	5 (4.3%)	0.098
Antioxidants	-	2 (1.7%)	0.300
Antipsychotics	-	-	-
Beta-blockers	9 (14.8%)	6 (5.2%)	0.031
Statins	25 (41.0%)	17 (14.8%)	<0.001
Hydrochlorothiazide	9 (14.8%)	2 (1.7%)	<0.001
Hydroxyurea	1 (1.6%)	-	0.168
ACE inhibitors (captopril, enalapril, and ramipril)	15 (24.6%)	7 (6.1%)	<0.001
Metformin	11 (18.0%)	6 (5.2%)	0.006
Omeprazole	24 (39.3%)	19 (16.5%)	<0.001
Vitamin D	8 (13.1%)	15 (13.0%)	0.989

Abbreviations: N, number of subjects; NSAIDs, non-steroidal anti-inflammatory drugs; ACE, angiotensin-converting enzyme.

**Table 3:** Sun exposure and photoprotection measures.

Variable		SCC	Control	*p*-Value
Outdoor sunbathing, days/year, *N* (%)	Never	29 (49.2%)	29 (23.0%)	0.057
	1–5 days	7 (11.9%)	19 (15.1%)	
	6–30 days	13 (22.0%)	53 (42.1%)	
	31–90 days	7 (11.9%)	21 (16.7%)	
	>90 days	3 (5.1%)	4 (3.2%)	
Days/year practicing outdoor sports, *N* (%)	Never	29 (50.9%)	31 (24.6%)	0.004
	1–5 days	5 (8.8%)	21 (16.7%)	
	6–30 days	6 (10.5%)	35 (27.8%)	
	31–90 days	6 (10.5%)	16 (12.7%)	
	>90 days	11 (19.3%)	23 (18.3%)	
Outdoor sunbathing, hours/day, *N* (%)	1–2 h	32 (78.0%)	76 (71.7%)	0.484
	3–4 h	6 (14.6%)	25 (23.6%)	
	5–6 h	3 (7.3%)	4 (3.8%)	
	>6 h	-	1 (0.9%)	
Hours/day practicing outdoor sport, *N* (%)	1–2 h	34 (81.0%)	92 (86.8%)	0.129
	3–4 h	6 (14.3%)	13 (12.3%)	
	5–6 h	2 (4.8%)	-	
	>6 h	-	1 (0.9%)	
Use of shade, *N* (%)	Never/Rarely	17 (29.8%)	28 (22.6%)	0.463
	Sometimes	9 (15.8%)	27 (21.8%)	
	Habitually/Always	31 (54.4%)	69 (55.6%)	
Use of sunglasses, *N* (%)	Never/Rarely	25 (42.4%)	36 (28.8%)	0.183
	Sometimes	8 (13.6%)	23 (18.4%)	
	Habitually/Always	26 (44.1%)	66 (52.8%)	
Use of hat or cap, *N* (%)	Never/Rarely	20 (33.9%)	67 (53.6%)	0.037
	Sometimes	13 (22.0%)	32 (25.6%)	
	Habitually/Always	26 (44.1%)	26 (20.8%)	
Use of clothes, *N* (%)	Never/Rarely	21 (35.6%)	49 (39.8%)	0.849
	Sometimes	21 (35.6%)	42 (34.1%)	
	Habitually/Always	17 (28.8%)	32 (26.0%)	
Sun exposure from 12:00 to 16:00, *N* (%)	Never/Rarely	10 (17.2%)	23 (18.7%)	0.817
	Sometimes	9 (15.5%)	23 (18.7%)	
	Habitually/Always	39 (67.2%)	77 (62.6%)	
Use of sunscreen, *N* (%)	Never/Rarely	18 (30.5%)	20 (16.3%)	0.052
	Sometimes	13 (22.0%)	24 (19.5%)	
	Habitually/Always	28 (47.5%)	79 (64.2%)	
15 years ago, you were more exposed to sunlight, *N* (%)	Yes	46 (79.3%)	78 (62.9%)	0.026
	No	12 (20.7%)	46 (37.1%)	
SPF used 15 years ago, *N* (%)	I do not know	31 (59.6%)	22 (18.2%)	<0.001
	2–10	5 (9.6%)	11 (9.1%)	
	11–20	2 (3.8%)	17 (14.0%)	
	21–50	9 (17.3%)	37 (30.6%)	
	>50	5 (9.6%)	34 (28.1%)	
SPF used now, *N* (%)	I do not know	11 (20.4%)	10 (8.3%)	0.242
	2–10	2 (3.7%)	3 (2.5%)	
	11–20	2 (3.7%)	6 (5.0%)	
	21–50	12 (22.2%)	30 (25.0%)	
	>50	27 (50.0%)	71 (59.2%)	

Abbreviations: N, number of subjects; SPF, Sun Protection Factor.

**Table 4:** Lifestyle- and stress-related variables in the study population.

Variable		SCC	Control	*p*-Value
Relaxation activities, *N* (%)	Yes	4 (6.7%)	29 (23.2%)	0.006
	No	56 (93.3%)	96 (76.8%)	
Sport, *N* (%)	Yes	27 (45.8%)	80 (65.0%)	0.013
	No	32 (54.2%)	43 (35.0%)	
Years practicing sport, *Mean* (*SD*) [*P25*;*P75*]		29.04 (21.40) [10.0; 40.0]	20.84 (16.14) [10.0; 30.0]	0.056
Location of sport, *N* (%)	Indoor	3 (11.1%)	21 (26.6%)	0.229
	Outdoor	20 (74.1%)	46 (58.2%)	
	Indoor/outdoor	4 (14.8%)	12 (15.2%)	
Hours/week *Mean* (*SD*) [*P25*;*P75*]		6.21 (3.73) [3.0; 8.0]	5.58 (3.00) [3.0; 7.0]	0.398
Hours/day screentime, *N* (%)	<1 h	40 (72.7%)	27 (22.1%)	<0.001
	1–2 h	8 (14.5%)	34 (27.9%)	
	>3 h	7 (12.7%)	61 (50.0%)	
Smoker, *N* (%)	Yes	6 (10.2%)	25 (21.9%)	0.002
	No	27 (45.8%)	66 (57.9%)	
	Former smoker	27 (44.1%)	23 (20.2%)	
Cigarettes/day, *Mean* (*SD*) [*P25*;*P75*]		4 (-) [4.0; 4.0]	8.86 (4.29) [6.0; 10.0]	0.294
Hours/day of sleep in the last 5 years, *N* (%)	<6 h	3 (5.1%)	13 (10,4%)	<0.001
	6 h	9 (15.3%)	24 (19.2%)	
	7 h	12 (20.3%)	54 (43.2%)	
	8 h	23 (39.0%)	33 (26.4%)	
	>10 h	12 (20.3%)	1 (0.8%)	
Perceived stress, * *Mean* (*SD*) [*P25*;*P75*]		17.37 (9.69) [10.0; 24.0]	19.69 (8.99) [14.0; 26.0]	0.139
Sunburns in the last year, *N* (%)	0	30 (63.8%)	78 (78.8%)	<0.001
	1	3 (6.4%)	14 (14.1%)	
	2	7 (14.9%)	7 (7.1%)	
	≥3	7 (14.9%)	-	

* Individual scores on the PSS can range from 0 to 40, with higher scores indicating higher perceived stress: scores ranging from 0 to 13 are considered low stress, from 14 to 26 moderate stress, and from 27 to 40 high stress. Abbreviations: N, number of subjects; P25, 25th percentile; P75, 75th percentile; SD, standard deviation.

**Table 5:** Logistic regression findings: variables significantly associated with the presence of SCC.

Variable	Coefficient	*p*-Value
Age	0.01651	<0.001
Hair color	0.10408	0.0047
Phototype	0.05189	0.0441
Current work place (indoors)	−0.47321	<0.001
Outside work previously	0.51092	<0.001
Daily exposure hours	0.07420	0.0513
Years of exposure	0.01041	0.0310
Use of hat or cap	0.09340	<0.001
15 years ago, were more exposed to ultraviolet radiation	0.17656	0.0229
Relaxation activities	−0.26786	0.0038
Hours/day with screens	−0.25152	<0.001
Smoker	0.17742	<0.001
Anxiolytics	0.20578	0.0289
Antidepressants	0.30937	0.0050
Beta-blockers	0.27702	0.0311
Statins	0.32658	<0.001
Hydrochlorothiazide	0.50303	<0.001
ACE inhibitors (captopril, enalapril, and ramipril)	0.38312	<0.001
Metformin	0.33259	0.0060
Omeprazole	0.27994	<0.001
Linolenic acid	0.04800	0.0419
Coffee	−0.03609	0.0133

Abbreviations: ACE, angiotensin-converting enzyme.

## Data Availability

The data presented in this study are available in this article (and Supplementary Material).

## Supplementary Materials

The following supporting information can be downloaded at https://www.mdpi.com/article/10.3390/cancers15225376/s1, Table S1: The dietary intake of nutrients calculated using the Predimed questionnaire.

## References

[1] Wild C.P. (2005). Complementing the Genome with an “Exposome”: The Outstanding Challenge of Environmental Exposure Measurement in Molecular Epidemiology. Cancer Epidemiol. Biomarkers Prev..

[2] Vineis P., Chadeau-Hyam M., Gmuender H., Gulliver J., Herceg Z., Kleinjans J., Kogevinas M., Kyrtopoulos S., Nieuwenhuijsen M., Phillips D. (2017). The exposome in practice: Design of the EXPOsOMICS project. Int. J. Hyg. Environ. Heal..

[3] Holterhues C., de Vries E., Louwman M.W., Koljenović S., Nijsten T. (2010). Incidence and Trends of Cutaneous Malignancies in the Netherlands, 1989–2005. J. Investig. Dermatol..

[4] Tejera-Vaquerizo A., Descalzo-Gallego M.A., Otero-Rivas M.M., Posada-García C., Rodríguez-Pazos L., Pastushenko I., Marcos-Gragera R., García-Doval I. (2016). Skin cancer incidence and mortality in Spain: A systematic review and meta-analysis. Actas Dermo-Sifiliogr..

[5] IARC (1992). Solar and ultraviolet radiation. IARC Monographs on the Evaluation of Carcinogenic Risks to Humans.

[6] Slominski A.T., Zmijewski M.A., Plonka P.M., Szaflarski J.P., Paus R. (2018). How UV Light Touches the Brain and Endocrine System Through Skin, and Why. Endocrinology.

[7] de Troya-Martín M., Blázquez-Sánchez N., Rivas-Ruiz F., Fernández-Canedo I., Rupérez-Sandoval A., Pons-Palliser J., Perea-Milla E. (2009). Validación de un cuestionario en español sobre comportamientos, actitudes y conocimientos relacionados con la exposición solar (Validation of a Spanish questionnaire to evaluate habits, attitudes, and understanding of exposure to sunlight: “the beach questionnaire”). Actas Dermosifiliogr..

[8] Gilaberte Y., Casanova J.M., García-Malinis A.J., Arias-Santiago S., de la Fuente M.R.G., Pamiés-Gracia M., Ramirez-Palomino J., Ruiz-Campos I., Gracia-Cazaña T., Buendia-Eisman A. (2020). Skin Cancer Prevalence in Outdoor Workers of Ski Resorts. J. Ski. Cancer.

[9] Ruiz-Canela M., Zazpe I., Shivappa N., Hébert J.R., Sánchez-Tainta A., Corella D., Salas-Salvadó J., Fitó M., Lamuela-Raventós R.M., Rekondo J. (2015). Dietary inflammatory index and anthropometric measures of obesity in a population sample at high cardiovascular risk from the PREDIMED (PREvención con DIeta MEDiterránea) trial. Br. J. Nutr..

[10] Cohen S., Kamarck T., Mermelstein R. (1983). A Global Measure of Perceived Stress. J. Heal. Soc. Behav..

[11] Remor E. (2006). Psychometric Properties of a European Spanish Version of the Perceived Stress Scale (PSS). Span. J. Psychol..

[12] Waldman A., Schmults C. (2019). Cutaneous Squamous Cell Carcinoma. Hematol. Oncol. Clin. North Am..

[13] Armstrong B.K., Kricker A. (2001). The epidemiology of UV induced skin cancer. J. Photochem. Photobiol. B Biol..

[14] Tokez S., Wakkee M., Louwman M., Noels E., Nijsten T., Hollestein L. (2020). Assessment of Cutaneous Squamous Cell Carcinoma (cSCC) In situ Incidence and the Risk of Developing Invasive cSCC in Patients with Prior cSCC In situ vs. the General Population in the Netherlands, 1989–2017. JAMA Dermatol..

[15] van der Leest R., Liu L., Coebergh J., Neumann H., Mooi W., Nijsten T., de Vries E. (2012). Risk of second primary in situ and invasive melanoma in a Dutch population-based cohort: 1989–2008. Br. J. Dermatol..

[16] Augustin J., Kis A., Sorbe C., Schäfer I., Augustin M. (2018). Epidemiology of skin cancer in the German population: Impact of socioeconomic and geographic factors. J. Eur. Acad. Dermatol. Venereol..

[17] Ofenloch R., Schuttelaar M., Svensson Å., Bruze M., Naldi L., Cazzaniga S., Elsner P., Gonçalo M., Diepgen T. (2019). Socioeconomic Status and the Prevalence of Skin and Atopic Diseases in Five European Countries. Acta Derm.-Venereol..

[18] Zink A., Tizek L., Schielein M., Böhner A., Biedermann T., Wildner M. (2018). Different outdoor professions have different risks—A cross-sectional study comparing non-melanoma skin cancer risk among farmers, gardeners and mountain guides. J. Eur. Acad. Dermatol. Venereol..

[19] Moan J., Grigalavicius M., Baturaite Z., Dahlback A., Juzeniene A. (2015). The relationship between UV exposure and incidence of skin cancer. Photodermatol. Photoimmunol. Photomed..

[20] Yoshinaga S., Hauptmann M., Sigurdson A.J., Doody M.M., Freedman D.M., Alexander B.H., Linet M.S., Ron E., Mabuchi K. (2005). Nonmelanoma skin cancer in relation to ionizing radiation exposure among U.S. radiologic technologists. Int. J. Cancer.

[21] Kelfkens G., de Gruijl F.R., van der Leun J.C. (1991). Tumorigenesis by short-wave ultraviolet A: Papillomas versus squamous cell carcinomas. Carcinogenesis.

[22] Shyong E.Q., Lu Y., Goldstein A., Lebwohl M., Wei H. (2003). Synergistic enhancement of H_2_O_2_ production in human epidermoid carcinoma cells by Benzo[a]pyrene and ultraviolet A radiation. Toxicol. Appl. Pharmacol..

[23] Blakely K.M., Drucker A.M., Rosen C.F. (2019). Drug-Induced Photosensitivity—An Update: Culprit Drugs, Prevention and Management. Drug Saf..

[24] Pandeya N., Olsen C., Thompson B., Dusingize J., Neale R., Green A., Whiteman D., the QSkin Study (2019). Aspirin and nonsteroidal anti-inflammatory drug use and keratinocyte cancers: A large population-based cohort study of skin cancer in Australia. Br. J. Dermatol..

[25] Elmets C.A., Ledet J.J., Athar M. (2014). Cyclooxygenases: Mediators of UV-Induced Skin Cancer and Potential Targets for Prevention. J. Investig. Dermatol..

[26] Dou Y.-L., Lin J.-P., Liu F.-E., Wang L.-Y., Shu H.-H., Jiang N., Xie Y., Duan Q. (2014). Midazolam inhibits the proliferation of human head and neck squamous carcinoma cells by downregulating p300 expression. Tumor Biol..

[27] Kinjo T., Kowalczyk P., Kowalczyk M., Walaszek Z., Slaga T.J., Hanausek M. (2010). Effects of desipramine on the cell cycle and apoptosis in Ca3/7 mouse skin squamous carcinoma cells. Int. J. Mol. Med..

[28] Zhao C., Zhang H., Zhou J., Liu Q., Lu Q., Zhang Y., Yu X., Wang S., Liu R., Pu Y. (2022). Metabolomic transition trajectory and potential mechanisms of N-nitrosomethylbenzylamine induced esophageal squamous cell carcinoma in rats. Ecotoxicol. Environ. Saf..

[29] Shibuya C.M., Tjioe K.C., Oliveira S.H.P., Bernabé D.G. (2022). Propranolol inhibits cell viability and expression of the pro-tumorigenic proteins Akt, NF-ĸB, and VEGF in oral squamous cell carcinoma. Arch. Oral Biol..

[30] Ali S., Xie T., Amit M., Batalla-Covello J. (2022). β-Adrenergic signaling in skin cancer. FASEB BioAdv..

[31] Yang K., Marley A., Tang H., Song Y., Tang J.Y., Han J. (2017). Statin use and non-melanoma skin cancer risk: A meta-analysis of randomized controlled trials and observational studies. Oncotarget.

[32] Kubatka P., Kruzliak P., Rotrekl V., Jelinkova S., Mladosievicova B. (2014). Statins in oncological research: From experimental studies to clinical practice. Crit. Rev. Oncol. Hematol..

[33] Alrashidi A., Rhodes L.E., Sharif J.C.H., Kreeshan F.C., Farrar M.D., Ahad T. (2020). Systemic drug photosensitivity—Culprits, impact and investigation in 122 patients. Photodermatol. Photoimmunol. Photomed..

[34] Goldstein M.R., Mascitelli L., Pezzetta F. (2009). The double-edged sword of statin immunomodulation. Int. J. Cardiol..

[35] Götzinger F., Reichrath J., Millenaar D., Lauder L., Meyer M.R., Böhm M., Mahfoud F. (2022). Photoinduced skin reactions of cardiovascular drugs—A systematic review. Eur. Hear. J. Cardiovasc. Pharmacother..

[36] Mehlan J., Ueberschaar J., Hagenström K., Garbe C., Spitzer M.S., Druchkiv V., Schuettauf F. (2022). The use of HCT and/or ACE inhibitors significantly increases the risk of non-melanotic skin cancer in the periocular region. Graefe’s Arch. Clin. Exp. Ophthalmol..

[37] Garrido P.M., Borges-Costa J. (2020). Hydrochlorothiazide treatment and risk of non-melanoma skin cancer: Review of the literature. Rev. Port. Cardiol..

[38] Haisma M.S., Greven N., Logendran M., Bos J., Vegt B.V., Horváth B., De Vos S., De Bock G.H., Hak E., Rácz E. (2023). Chronic Use of Hydrochlorothiazide and Risk of Skin Cancer in Caucasian Adults: A PharmLines Initiative Inception Cohort Study. Acta Derm.-Venereol..

[39] Crist M., Yaniv B., Palackdharry S., Lehn M.A., Medvedovic M., Stone T., Gulati S., Karivedu V., Borchers M., Fuhrman B. (2022). Metformin increases natural killer cell functions in head and neck squamous cell carcinoma through CXCL1 inhibition. J. Immunother. Cancer.

[40] Chang M.S., Hartman R.I., Xue J., Giovannucci E.L., Nan H., Yang K. (2021). Risk of Skin Cancer Associated with Metformin Use: A Meta-Analysis of Randomized Controlled Trials and Observational Studies. Cancer Prev. Res..

[41] Qasem A., Kasabri V., AbuRish E., Bustanji Y., Al-Hiari Y., Al-Abbasi R., Abu-Irmaileh B., Alalawi S. (2020). The Evaluation of Potential Cytotoxic Effect of Different Proton Pump Inhibitors on Different Human Cancer Cell Lines. Anti-Cancer Agents Med. Chem..

[42] Backes C., Religi A., Moccozet L., Vuilleumier L., Vernez D., Bulliard J. (2018). Facial exposure to ultraviolet radiation: Predicted sun protection effectiveness of various hat styles. Photodermatol. Photoimmunol. Photomed..

[43] Black H.S., Rhodes L.E. (2016). Potential Benefits of Omega-3 Fatty Acids in Non-Melanoma Skin Cancer. J. Clin. Med..

[44] Black H.S., Rhodes L.E. (2006). The potential of omega-3 fatty acids in the prevention of non-melanoma skin cancer. Cancer Detect. Prev..

[45] Noel S.E., Stoneham A.C., Olsen C.M., Rhodes L.E., Green A.C. (2013). Consumption of omega-3 fatty acids and the risk of skin cancers: A systematic review and meta-analysis. Int. J. Cancer.

[46] Oh C.C., Jin A., Yuan J.-M., Koh W.-P. (2019). Coffee, tea, caffeine, and risk of nonmelanoma skin cancer in a Chinese population: The Singapore Chinese Health Study. J. Am. Acad. Dermatol..

[47] Fan F.S. (2022). Inhibition of NLRP3 inflammasome activation by caffeine might be a potential mechanism to reduce the risk of squamous cell carcinoma of the oral cavity and oropharynx with coffee drinking. Front. Oral Health.

[48] Rigel D.S. (2008). Cutaneous ultraviolet exposure and its relationship to the development of skin cancer. J. Am. Acad. Dermatol..

[49] Dozier S., Wagner R.F., Black S.A., Terracina J. (1997). Beachfront Screening for Skin Cancer in Texas Gulf Coast Surfers. South Med. J..

[50] Moehrle M. (2008). Outdoor sports and skin cancer. Clin. Dermatol..

[51] Yu M., King B., Ewert E., Su X., Mardiyati N., Zhao Z., Wang W. (2016). Exercise Activates p53 and Negatively Regulates IGF-1 Pathway in Epidermis within a Skin Cancer Model. PLoS ONE.

[52] Dhabhar F.S. (2013). Psychological stress and immunoprotection versus immunopathology in the skin. Clin. Dermatol..

[53] Pavlou P., Rallis M., Deliconstantinos G., Papaioannou G., Grando S. (2009). In-vivo data on the influence of tobacco smoke and UV light on murine skin. Toxicol. Ind. Heal..

[54] Leonardi-Bee J., Ellison T., Bath-Hextall F. (2012). Smoking and the Risk of Nonmelanoma Skin Cancer: Systematic review and meta-analysis. Arch. Dermatol..

[55] Sauvaigo S., Bonnet-Duquennoy M., Odin F., Hazane-Puch F., Lachmann N., Bonté F., Kurfürst R., Favier A. (2007). DNA repair capacities of cutaneous fibroblasts: Effect of sun exposure, age and smoking on response to an acute oxidative stress. Br. J. Dermatol..

[56] Curtin G.M., Hanausek M., Walaszek Z., Mosberg A.T., Slaga T.J. (2004). Short-Term In Vitro and In Vivo Analyses for Assessing the Tumor-Promoting Potentials of Cigarette Smoke Condensates. Toxicol. Sci..

