# Supplementary material for: The Influence of the Exposome in the Cutaneous Squamous Cell Carcinoma, a Multicenter Case–Control Study
Source: Cancers (Basel). 2023 Nov 12;15(22):5376. doi: 10.3390/cancers15225376 (PMC10670280; doi:10.3390/cancers15225376)
Supplement: Supplementary file 1 [file cancers-15-05376-s001.zip › cancers-2676272-supplementary.pdf]

Supplementary Material, Table 1. The dietary intake of nutrients calculated using the Predimed questionnaire

Variable		SCC	Control	p-value	Variable		SCC	Control	p-value
Whole milk	N	51	111	0.639	Carbohydrates	N	61	115	0.341
	Mean (SD)	1.29 (2.08)	1.13 (2.13)			Mean (SD)	249.35 (122.10)	231.49 (115.98)	
	Median	0.0	0.0			Median	224.2	211.3	
	(P25; P75)	(0.0; 3.0)	(0.0; 1.0)			(P25; P75)	(173.8; 286.5)	(155.9; 288.5)	
	(Min; Max)	(0.0; 6.0)	(0.0; 8.0)			(Min; Max)	(0.0; 655.2)	(0.0; 703.9)	
	N missing	10	4			N missing	0	0	
Semi-skimmed milk	N	55	107	0.177	Proteins	N	61	115	0.418
	Mean (SD)	2.60 (2.43)	2.06 (2.41)			Mean (SD)	114.84 (62.74)	104.80 (85.06)	
	Median	3.0	0.0			Median	102.3	93.1	
	(P25; P75)	(0.0; 5.0)	(0.0; 5.0)			(P25; P75)	(82.8; 125.1)	(75.3; 120.2)	
	(Min; Max)	(0.0; 6.0)	(0.0; 7.0)			(Min; Max)	(0.0; 422.1)	(0.0; 898.8)	
	N missing	6	8			N missing	0	0	
Skimmed milk	N	49	105	0.991	Fats	N	61	115	0.487
	Mean (SD)	1.20 (2.21)	1.20 (2.10)			Mean (SD)	104.17 (65.94)	96.93 (65.52)	
	Median	0.0	0.0			Median	88.1	86.0	
	(P25; P75)	(0.0; 0.0)	(0.0; 3.0)			(P25; P75)	(66.1; 112.1)	(64.2; 112.6)	
	(Min; Max)	(0.0; 6.0)	(0.0; 6.0)			(Min; Max)	(0.0; 345.9)	(0.0; 628.7)	
	N missing	12	10			N missing	0	0	
Egg	N	58	110	0.776	Monounsaturated fats	N	61	115	0.587
	Mean (SD)	2.79 (0.77)	2.75 (0.87)			Mean (SD)	43.84 (28.10)	41.50 (26.70)	
	Median	3.0	3.0			Median	38.6	39.3	
	(P25; P75)	(2.0; 3.0)	(2.0; 3.0)			(P25; P75)	(26.0; 48.6)	(27.2; 49.7)	
	(Min; Max)	(0.0; 5.0)	(0.0; 6.0)			(Min; Max)	(0.0; 143.4)	(0.0; 234.4)	
	N missing	3	5			N missing	0	0	
White fish	N	59	112	0.312	Polyunsaturated fats	N	61	115	0.205
	Mean (SD)	2.44 (1.10)	2.26 (1.12)			Mean (SD)	18.20 (17.75)	15.51 (10.38)	
	Median	3.0	2.0			Median	12.0	13.4	
	(P25; P75)	(2.0; 3.0)	(2.0; 3.0)			(P25; P75)	(9.1; 21.4)	(9.1; 19.3)	
	(Min; Max)	(0.0; 6.0)	(0.0; 6.0)			(Min; Max)	(0.0; 115.8)	(0.0; 87.0)	

	N missing	2	3			N missing	0	0	
Blue fish	N	57	109	0.672	Saturated fats	N	61	115	0.423
	Mean (SD)	1.95 (1.04)	1.87 (1.12)			Mean (SD)	32.33 (20.76)	29.65 (21.22)	
	Median	2.0	2.0			Median	26.9	25.5	
	(P25; P75)	(1.0; 3.0)	(1.0; 2.0)			(P25; P75)	(21.7; 37.4)	(17.8; 35.8)	
	(Min; Max)	(0.0; 4.0)	(0.0; 6.0)			(Min; Max)	(0.0; 141.1)	(0.0; 192.1)	
	N missing	4	6			N missing	0	0	
Almond	N	58	109	0.214	Alcohol	N	61	115	0.268
	Mean (SD)	1.43 (1.77)	1.74 (1.40)			Mean (SD)	8.86 (10.36)	7.01 (10.59)	
	Median	1.0	2.0			Median	4.4	3.4	
	(P25; P75)	(0.0; 2.0)	(1.0; 3.0)			(P25; P75)	(0.0; 11.8)	(0.0; 8.3)	
	(Min; Max)	(0.0; 8.0)	(0.0; 5.0)			(Min; Max)	(0.0; 46.6)	(0.0; 68.5)	
	N missing	3	6			N missing	0	0	
Walnut	N	58	107	0.675	Energy	N	61	115	0.340
	Mean (SD)	1.72 (1.95)	1.61 (1.56)			Mean (SD)	2456.24 (1169.62)	2266.66 (1294.28)	
	Median	1.0	1.0			Median	2149.6	2106.4	
	(P25; P75)	(0.0; 2.0)	(0.0; 3.0)			(P25; P75)	(1725.8; 2805.0)	(1551.1; 2838.7)	
	(Min; Max)	(0.0; 8.0)	(0.0; 5.0)			(Min; Max)	(0.0; 6864.7)	(0.0; 12397.4)	
	N missing	3	8			N missing	0	0	
Legumes (lentils, beans, chickpeas. peas)	N	60	109	0.118	Cholesterol	N	61	115	0.872
	Mean (SD)	1.48 (0.64)	1.32 (0.65)			Mean (SD)	524.41 (468.04)	506.62 (791.18)	
	Median	1.5	1.3			Median	411.6	405.1	
	(P25; P75)	(0.9; 2.0)	(0.8; 1.8)			(P25; P75)	(338.2; 601.2)	(318.3; 526.6)	
	(Min; Max)	(0.5; 3.0)	(0.0; 3.0)			(Min; Max)	(0.0; 3598.1)	(0.0; 8603.0)	
	N missing	1	6			N missing	0	0	
Olive oil	N	59	109	0.911	Fiber	N	61	115	0.082
	Mean (SD)	3.60 (1.62)	3.57 (1.54)			Mean (SD)	29.87 (16.66)	25.67 (14.39)	
	Median	3.0	3.0			Median	26.3	23.8	
	(P25; P75)	(2.5; 5.0)	(2.5; 5.0)			(P25; P75)	(18.7; 39.4)	(16.7; 34.2)	
	(Min; Max)	(0.5; 8.0)	(0.0; 8.0)			(Min; Max)	(0.0; 95.6)	(0.0; 99.1)	
	N missing	2	6			N missing	0	0	

Chocolate	N	58	106	0.510	Glycemic load	N	61	115	0.250
	Mean (SD)	1.31 (1.59)	1.47 (1.44)			Mean (SD)	122.34 (65.06)	111.33 (57.58)	
	Median	1.0	1.0			Median	108.6	104.5	
	(P25; P75)	(0.0; 2.0)	(0.0; 3.0)			(P25; P75)	(81.9; 136.4)	(70.1; 143.8)	
	(Min; Max)	(0.0; 6.0)	(0.0; 6.0)			(Min; Max)	(0.0; 330.4)	(0.0; 296.2)	
	N missing	3	9			N missing	0	0	
Decaffeinated coffee	N	55	108	0.134	Glycemic index of foods	N	60	113	0.283
	Mean (SD)	2.13 (2.23)	1.57 (2.22)			Mean (SD)	48.48 (4.51)	47.61 (5.38)	
	Median	1.0	0.0			Median	48.5	47.4	
	(P25; P75)	(0.0; 4.0)	(0.0; 3.5)			(P25; P75)	(45.7; 51.3)	(44.3; 51.8)	
	(Min; Max)	(0.0; 6.0)	(0.0; 6.0)			(Min; Max)	(38.7; 64.8)	(32.3; 58.2)	
	N missing	6	7			N missing	1	2	
Caffeinated coffee	N	54	107	0.013	Iron	N	61	115	0.452
	Mean (SD)	2.50 (2.34)	3.55 (2.60)			Mean (SD)	19.59 (9.65)	17.98 (15.13)	
	Median	2.0	5.0			Median	17.4	15.6	
	(P25; P75)	(0.0; 5.0)	(0.0; 6.0)			(P25; P75)	(13.5; 22.3)	(12.2; 20.8)	
	(Min; Max)	(0.0; 6.0)	(0.0; 8.0)			(Min; Max)	(0.0; 57.5)	(0.0; 156.9)	
	N missing	7	8			N missing	0	0	
Tea	N	54	107	0.096	Sodium	N	61	115	0.451
	Mean (SD)	0.76 (1.49)	1.29 (2.07)			Mean (SD)	2730.10 (2265.04)	2492.70 (1823.30)	
	Median	0.0	0.0			Median	2302.5	2233.7	
	(P25; P75)	(0.0; 1.0)	(0.0; 2.0)			(P25; P75)	(1751.5; 2830.4)	(1671.4; 2925.2)	
	(Min; Max)	(0.0; 6.0)	(0.0; 7.0)			(Min; Max)	(0.0; 15696.8)	(0.0; 17631.1)	
	N missing	7	8			N missing	0	0	
Wine	N	57	111	0.086	Magnesium	N	61	115	0.234
	Mean (SD)	1.20 (1.44)	0.86 (1.06)			Mean (SD)	459.70 (236.81)	414.31 (242.10)	
	Median	1.0	0.7			Median	390.2	376.2	
	(P25; P75)	(0.0; 1.7)	(0.0; 1.3)			(P25; P75)	(334.1; 527.4)	(287.2; 494.6)	
	(Min; Max)	(0.0; 6.0)	(0.0; 5.0)			(Min; Max)	(0.0; 1444.7)	(0.0; 2250.1)	
	N missing	4	4			N missing	0	0	
Vitamin A	N	61	115	0.247	Potassium	N	61	115	0.221

	Mean (SD)	2500.69 (4716.80)	1759.38 (3620.51)			Mean (SD)	5110.31 (2293.99)	4626.16 (2588.76)	
	Median	1447.5	1299.4			Median	4687.9	4269.5	
	(P25; P75)	(901.4; 2104.3)	(794.3; 1791.3)			(P25; P75)	(3814.5; 5939.6)	(3301.6; 5678.1)	
	(Min; Max)	(0.0; 36279.9)	(0.0; 38725.0)			(Min; Max)	(0.0; 13874.7)	(0.0; 22714.6)	
	N missing	0	0			N missing	0	0	
Vitamin D	N	61	115	0.934	Iodine	N	61	115	0.883
	Mean (SD)	7.27 (5.74)	7.39 (10.44)			Mean (SD)	297.19 (158.47)	292.20 (238.45)	
	Median	5.2	5.2			Median	274.5	255.3	
	(P25; P75)	(3.4; 10.2)	(3.7; 8.7)			(P25; P75)	(202.7; 396.4)	(150.3; 341.6)	
	(Min; Max)	(0.0; 29.4)	(0.0; 101.0)			(Min; Max)	(0.0; 648.2)	(0.0; 1727.1)	
	N missing	0	0			N missing	0	0	
Vitamin E	N	61	115	0.785	Selenium	N	61	115	0.439
	Mean (SD)	10.38 (6.49)	10.73 (8.62)			Mean (SD)	112.38 (73.85)	100.98 (101.59)	
	Median	8.6	9.4			Median	92.6	84.2	
	(P25; P75)	(6.8; 12.0)	(7.2; 13.1)			(P25; P75)	(70.3; 133.0)	(64.3; 115.4)	
	(Min; Max)	(0.0; 37.1)	(0.0; 86.9)			(Min; Max)	(0.0; 367.7)	(0.0; 1049.6)	
	N missing	0	0			N missing	0	0	
Vitamin C	N	61	115	0.918	Zinc	N	61	115	0.337
	Mean (SD)	234.06 (139.65)	231.44 (170.42)			Mean (SD)	13.99 (7.74)	12.52 (10.52)	
	Median	210.7	216.0			Median	11.7	11.0	
	(P25; P75)	(128.3; 288.4)	(136.5; 287.9)			(P25; P75)	(10.1; 15.3)	(9.1; 14.2)	
	(Min; Max)	(0.0; 552.4)	(0.0; 1408.2)			(Min; Max)	(0.0; 48.2)	(0.0; 112.3)	
	N missing	0	0			N missing	0	0	
Vitamin B1	N	61	115	0.573	Phosphorus	N	61	115	0.487
	Mean (SD)	2.46 (1.12)	2.32 (1.74)			Mean (SD)	2015.22 (1078.35)	1881.53 (1277.20)	
	Median	2.3	2.0			Median	1861.4	1684.3	
	(P25; P75)	(1.7; 3.1)	(1.4; 2.7)			(P25; P75)	(1460.6; 2159.7)	(1310.7; 2115.5)	
	(Min; Max)	(0.0; 6.1)	(0.0; 13.0)			(Min; Max)	(0.0; 6169.9)	(0.0; 12218.5)	
	N missing	0	0			N missing	0	0	
Vitamin B2	N	61	115	0.482	Trans fatty acids	N	61	115	0.647
	Mean (SD)	2.49 (1.74)	2.28 (1.92)			Mean (SD)	0.74 (0.62)	0.78 (0.67)	

	Median	2.2	1.9			Median	0.6	0.6	
	(P25; P75)	(1.7; 2.8)	(1.5; 2.5)			(P25; P75)	(0.4; 0.9)	(0.4; 0.9)	
	(Min; Max)	(0.0; 13.0)	(0.0; 18.7)			(Min; Max)	(0.0; 4.0)	(0.0; 4.6)	
	N missing	0	0			N missing	0	0	
Vitamin B3	N	61	115	0.501	Beta Carotene	N	61	115	0.552
	Mean (SD)	49.83 (28.90)	45.86 (40.88)			Mean (SD)	3692.55 (3595.65)	3403.05 (2750.89)	
	Median	43.5	40.2			Median	2270.4	2371.6	
	(P25; P75)	(34.8; 58.5)	(31.4; 51.4)			(P25; P75)	(1527.6; 5315.2)	(1717.5; 4692.6)	
	(Min; Max)	(0.0; 209.6)	(0.0; 435.1)			(Min; Max)	(0.0; 22554.9)	(0.0; 13653.4)	
	N missing	0	0			N missing	0	0	
Vitamin B6	N	61	115	0.303	Omega-3	N	61	115	0.975
	Mean (SD)	2.88 (1.43)	2.59 (1.91)			Mean (SD)	1.10 (0.82)	1.10 (1.80)	
	Median	2.5	2.3			Median	0.8	0.8	
	(P25; P75)	(2.0; 3.3)	(1.8; 2.9)			(P25; P75)	(0.5; 1.5)	(0.5; 1.2)	
	(Min; Max)	(0.0; 8.0)	(0.0; 18.6)			(Min; Max)	(0.0; 3.9)	(0.0; 17.9)	
	N missing	0	0			N missing	0	0	
Folic Acid	N	61	115	0.219	Omega-6	N	61	115	0.199
	Mean (SD)	445.30 (229.85)	396.36 (260.66)			Mean (SD)	13.84 (14.42)	11.69 (7.79)	
	Median	392.0	341.9			Median	9.6	10.0	
	(P25; P75)	(287.5; 565.4)	(251.9; 468.3)			(P25; P75)	(7.0; 16.4)	(6.6; 15.4)	
	(Min; Max)	(0.0; 1115.4)	(0.0; 2047.3)			(Min; Max)	(0.0; 98.2)	(0.0; 60.6)	
	N missing	0	0			N missing	0	0	
Vitamin B12	N	61	115	0.354	White meat	N	61	115	0.575
	Mean (SD)	17.01 (31.86)	12.39 (31.12)			Mean (SD)	79.28 (93.96)	71.09 (91.00)	
	Median	10.5	8.6			Median	63.0	63.0	
	(P25; P75)	(6.8; 14.2)	(5.5; 12.4)			(P25; P75)	(31.1; 105.0)	(21.0; 73.0)	
	(Min; Max)	(0.0; 245.9)	(0.0; 336.1)			(Min; Max)	(0.0; 642.0)	(0.0; 750.0)	
	N missing	0	0			N missing	0	0	
Calcium	N	61	115	0.605	Processed meats, g/person/day	N	61	115	0.802
	Mean (SD)	1125.62 (563.70)	1075.23 (640.86)			Mean (SD)	33.80 (41.03)	35.77 (53.53)	
	Median	1076.8	956.4			Median	25.2	25.2	
	(P25; P75)	(782.0; 1338.9)	(702.2; 1300.5)			(P25; P75)	(15.4; 33.6)	(14.4; 46.0)	

	(Min; Max)	(0.0; 3320.4)	(0.0; 4548.0)			(Min; Max)	(0.0; 300.0)	(0.0; 526.7)	
	N missing	0	0			N missing	0	0	
Arachidonic acid	N	61	115	0.562	Red meat	N	61	115	0.271
	Mean (SD)	0.22 (0.15)	0.20 (0.26)			Mean (SD)	92.35 (98.66)	72.17 (123.40)	
	Median	0.2	0.2			Median	69.7	42.0	
	(P25; P75)	(0.1; 0.3)	(0.1; 0.2)			(P25; P75)	(42.0; 94.0)	(21.0; 94.0)	
	(Min; Max)	(0.0; 1.1)	(0.0; 2.7)			(Min; Max)	(0.0; 647.0)	(0.0; 1250.0)	
	N missing	0	0			N missing	0	0	
Phytosterols	N	61	115	0.412	Retinol	N	61	115	0.266
	Mean (SD)	368.81 (224.40)	342.57 (188.23)			Mean (SD)	1353.31 (4664.00)	672.65 (3357.83)	
	Median	306.4	300.6			Median	245.4	226.6	
	(P25; P75)	(215.4; 427.6)	(229.3; 443.6)			(P25; P75)	(127.8; 453.9)	(141.8; 388.2)	
	(Min; Max)	(0.0; 1132.5)	(0.0; 1234.7)			(Min; Max)	(0.0; 35446.9)	(0.0; 36048.1)	
	N missing	0	0			N missing	0	0	
Linoleic Acid	N	61	115	0.291	Lactose	N	61	115	0.805
	Mean (SD)	14.47 (15.28)	12.58 (8.38)			Mean (SD)	15.25 (10.86)	14.71 (15.21)	
	Median	9.8	10.8			Median	12.8	11.4	
	(P25; P75)	(7.0; 16.6)	(7.0; 15.5)			(P25; P75)	(9.1; 20.0)	(6.4; 19.8)	
	(Min; Max)	(0.0; 100.2)	(0.0; 60.6)			(Min; Max)	(0.0; 50.9)	(0.0; 126.3)	
	N missing	0	0			N missing	0	0	
Linolenic Acid	N	61	115	0.041					
	Mean (SD)	1.89 (2.25)	1.40 (0.92)						
	Median	1.2	1.2						
	(P25; P75)	(0.9; 1.8)	(0.8; 1.8)						
	(Min; Max)	(0.0; 14.9)	(0.0; 6.7)						
	N missing	0	0						

Abbreviations: SCC, Squamous Cell Carcinoma; N, number of subjects; N missing, number of missing observations; P25, 25th percentile; P75, 75th percentile; SD, standard deviation

SUPPLEMENTARY MATERIAL 1

QUESTIONNAIRES

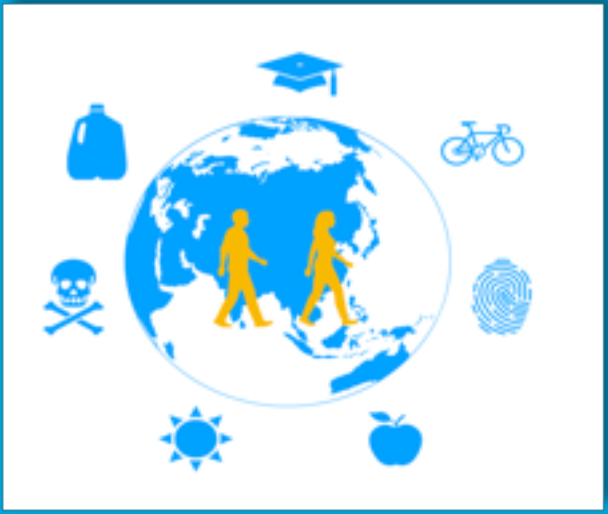

Exposoma
y
Cáncer de Piel

Identificación:

1.- Sexo:

2.- Edad:

3.- Estado Civil:

- o Soltero
- o Casado/a o convivencia en pareja
- o Viudo/a
- o Separado/a o divorciado/a

4. Ingresos Anuales :____________________€

5. Talla:__________cm

6. Peso:__________Kg

7. Provincia de residencia:___________

a. Entorno de residencia:

- o Rural
- o Urbano

b. Municipio:_______________

8. PROFESIÓN

a. Cuál es su profesión:

Profesión actual (en relación con exposición solar):
- o Interior
- o Exterior

* Si exterior: número de horas de exposición solar diarias________horas

* Si interior: ¿cerca de una ventana?
- o Sí
- o No

b. Si alguna profesión previa con exposición al sol:

Nº de horas de exposición solar diarias__________h

Nº de años trabajando expuesto al sol_______años

c. Trabaja expuesto a algún tipo de sustancia tóxica:
- o Sí

Si afirmativo, señale cuál:
- Pesticidas
- Arsénico
- Coal tar
- Antracenos
- Parafinas
- Asfalto
- Aceites minerales
- Petróleo
- Otros:

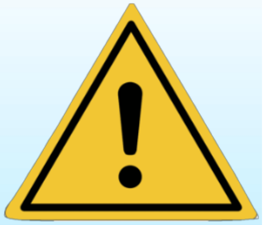

d. Trabaja expuesto a radiaciones ionizantes:
- o Sí
- o No

e. Trabaja como piloto o tripulación de avión
- o Sí
- o No

Si afirmativo, Nº de horas/ semanales ☐

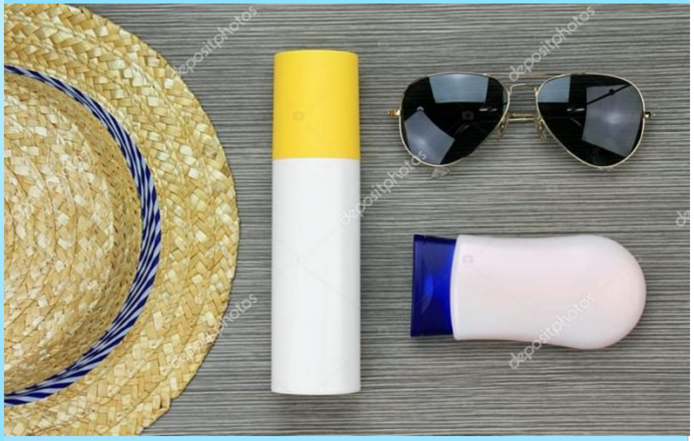

9. HÁBITOS DE EXPOSICIÓN SOLAR:

**¿Cuántos días al año se expone al sol realizando actividades al aire libre?**
Baños de sol (playa, piscina, solárium): *Ningún día*☐ *1-5 días*☐ *6-30 días*☐ *31-90 días*☐ *Más de 90 días*☐
Deportes al aire libre: *Ningún día*☐ *1-5 días*☐ *6-30 días*☐ *31-90 días*☐ *Más de 90 días*☐
Trabajos al aire libre: *Ningún día*☐ *1-5 días*☐ *6-30 días*☐ *31-90 días*☐ *Más de 90 días*☐

**¿Cuántas horas al día se suele exponer al sol realizando actividades al aire libre?**
Baños de sol (playa, piscina, solárium): *1-2 horas* ☐ *3-4 horas*☐ *5-6 horas*☐ *Más de 6 horas*☐
Deportes al aire libre: *1-2 horas* ☐ *3-4 horas*☐ *5-6 horas*☐ *Más de 6 horas*☐
Trabajos al aire libre: *1-2 horas* ☐ *3-4 horas*☐ *5-6 horas*☐ *Más de 6 horas*☐

10. PRÁCTICAS DE PROTECCIÓN SOLAR:

Medidas:
Me resguardo a la sombra / Uso sombrilla: *Nunca* ☐ *Casi nunca* ☐ *A veces* ☐ *Casi siempre* ☐ *Siempre* ☐
Uso gafas de sol: *Nunca* ☐ *Casi nunca* ☐ *A veces* ☐ *Casi siempre* ☐ *Siempre* ☐
Uso sombrero o gorra: *Nunca* ☐ *Casi nunca* ☐ *A veces* ☐ *Casi siempre* ☐ *Siempre* ☐
Uso camiseta con manga y pantalón largo: *Nunca* ☐ *Casi nunca* ☐ *A veces* ☐ *Casi siempre* ☐ *Siempre* ☐
Evito las horas del mediodía (12:00 a 16:00 h): *Nunca* ☐ *Casi nunca* ☐ *A veces* ☐ *Casi siempre* ☐ *Siempre* ☐
Uso cremas de protección solar: *Nunca* ☐ *Casi nunca* ☐ *A veces* ☐ *Casi siempre* ☐ *Siempre* ☐

**Cuando utiliza cremas de protección solar, ¿Qué índice de protección solar suele usar?**
No lo sé ☐ *2-10* ☐ *11-20* ☐ *21-50* ☐ *mas de 50* ☐

a. Hace más de 15 años:

¿Estaba expuesto al sol más que en la actualidad?

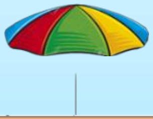

- o Sí
- o No

**Cuando utilizaba cremas de protección solar, ¿Qué índice de protección solía usar?**
No lo sé ☐ *2-10* ☐ *11-20* ☐ *21-50* ☐ *mas de 50* ☐

11. VARIABLES CUTÁNEAS

a. Color de pelo:

- o Pelirrojo
- o Rubio
- o Castaño claro
- o Castaño oscuro
- o Negro

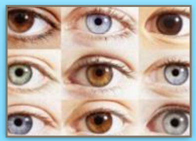

b. Color de ojos:

- o Azules
- o Verdes
- o Verdes oscuros/pardos
- o Marrones claros
- o Marrones

[illegible]

Fototipo I	Fototipo II	Fototipo III	Fototipo IV	Fototipo V	Fototipo VI
Siempre se quema y nunca se broncea.	Casi siempre se quema y a veces se broncea.	A veces se quema y generalmente se broncea.	Raro que se queme y siempre se broncea.	Se quema raramente. Bronceado muy intenso.	Casi nunca se quema.
Ojos y pelo claros. Piel muy blanca.	Ojos azules o pardos. Pelo rubio o pelirrojo. Piel blanca.	Tipo nórdico alpino.	Ojos y pelo oscuros. Piel blanca o un poco tostada.	Piel con un tono marrón.	Razas negras.

11.-VARIABLES DEL ESTILO DE VIDA:

a. Realiza actividades de relajación:

- o Meditación
- o Yoga
- o Mindfulness
- o Yoga
- o Otros
- o Nada

b. Actividad deportiva:

Realiza ejercicio:

- o Sí
- o No

Si afirmativo: Hace cuántos años?

Si afirmativo: I

- o Interior
- o Exterior
- o Ambos

Si afirmativo, Nº de horas/ semanales ☐

c. Tabaquismo:

o *Es fumador actual:*

- o Sí
- o No

Si afirmativo, Nº de cigarrillos/día ☐

o *Es exfumador:*

Tiempo transcurrido desde que lo dejó

Consumo cuando lo dejó Nº de cigarrillos/ día ☐

o *No fumador*

d. Sueño:

Horas de sueño/día en los últimos 5

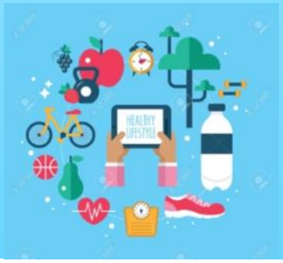

- o <5 horas
- o <7 horas
- o ≥7 horas

12. ESTRES PERCIBIDO

	Nunca	Casi nunca	De vez en cuando	A menudo	Muy a menudo
1. En el último mes, ¿con qué frecuencia ha estado afectado por algo que ha ocurrido inesperadamente?	0	1	2	3	4
2. En el último mes, ¿con qué frecuencia se ha sentido incapaz de controlar las cosas importantes en su vida?	0	1	2	3	4
3. En el último mes, ¿con qué frecuencia se ha sentido nervioso o estresado?	0	1	2	3	4
4. En el último mes, ¿con qué frecuencia ha manejado con éxito los pequeños problemas irritantes de la vida?	0	1	2	3	4
5. En el último mes, ¿con qué frecuencia ha sentido que ha afrontado efectivamente los cambios importantes que han estado ocurriendo en su vida?	0	|1	2	3	4
6. En el último mes, ¿con qué frecuencia ha estado seguro sobre su capacidad para manejar sus problemas personales?	0	1	2	3	4
7. En el último mes, ¿con qué frecuencia ha sentido que las cosas le van bien?	0	1	2	3	4
8. En el último mes, ¿con qué frecuencia ha sentido que no podía afrontar todas las cosas que tenía que hacer?	0	1	2	3	4
9. En el último mes, ¿con qué frecuencia ha podido controlar las dificultades de su vida?	0	1	2	3	4
10. En el ultimo mes, ¿con que frecuencia se ha sentido que tenia todo bajo control?	0	1	2	3	4
11. En el último mes, ¿con qué frecuencia ha estado enfadado porque las cosas que le han ocurrido estaban fuera de su control?	0	1	2	3	4
12. En el último mes, ¿con qué frecuencia ha pensado sobre las cosas que le quedan por hacer?	0	1	2	3	4
13. En el último mes, ¿con qué frecuencia ha podido controlar la forma de pasar el tiempo?	0	1	2	3	4
14. En el último mes, ¿con qué frecuencia ha sentido que las dificultades se acumulan tanto que no puede superarlas?	0	1	2	3	4

Las preguntas en esta escala hacen referencia a sus sentimientos y pensamientos durante el **último mes**. En cada caso, por favor indique con una "X" cómo usted se ha sentido o ha pensado en cada situación.

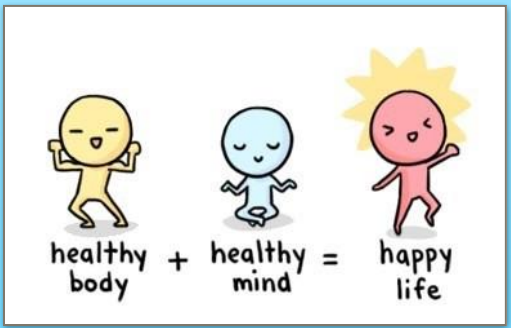

IDENTIFICACIÓN DEL PARTICIPANTE

NODO
01. Andalucía-Málaga
02. Andalucía-Sevilla-San Pablo
03. Andalucía-Sevilla-V. Rocío
04. Baleares
05. Catalunya-Barna Norte
06. Catalunya-Barna Sur
07. Catalunya-Reus-Tarragona
08. Madrid Norte
09. Madrid Sur
10. Navarra
11. País Vasco
12. Valencia

marque así

así no marque

NODO		CENTRO		MÉDICO		PACIENTE		VISITA	
0	0	0	0	0	0	0	0	0	0
1	1	1	1	1	1	1	1	1	1
2	2	2	2	2	2	2	2	2	2
3	3	3	3	3	3	3	3	3	3
4	4	4	4	4	4	4	4	4	4
5	5	5	5	5	5	5	5	5	5
6	6	6	6	6	6	6	6	6	6
7	7	7	7	7	7	7	7	7	7
8	8	8	8	8	8	8	8	8	8
9	9	9	9	9	9	9	9	9	9

Por favor, marque una única opción para cada alimento.

Para cada alimento, marque el recuadro que indica la frecuencia de consumo **por término medio** durante el **año pasado**. Se trata de tener en cuenta también la variación verano/invierno. Por ejemplo, si toma helados 4 veces/semana sólo durante los 3 meses de verano, el uso promedio al año es 1/semana

I. LACTEOS

	CONSUMO MEDIO DURANTE EL AÑO PASADO								
	NUNCA O CASI NUNCA	AL MES	A LA SEMANA			AL DÍA			
		1 - 3	1	2 - 4	5 - 6	1	2 - 3	4 - 6	6 +
1. Leche entera (1 taza, 200 cc)									
2. Leche semidesnatada (1 taza, 200 cc)									
3. Leche descremada (1 taza, 200 cc)									
4. Leche condensada (1 cucharada)									
5. Nata o crema de leche (1/2 taza)									
6. Batidos de leche (1 vaso, 200 cc)									
7. Yogurt entero (1, 125 gr.)									
8. Yogurt descremado (1, 125 gr.)									
9. Petit suisse (1, 55 gr.)									
10. Requesón o cuajada (1/2 taza)									
11. Queso en porciones o cremoso (1, porción 25 gr.)									
12. Otros quesos: curados, semicurados (Manchego, Bola, Emmental…) (50 gr.)									
13. Queso blanco o fresco (Burgos, cabra…) (50 gr.)									
14. Natillas, flan, puding (1, 130 cc)									
15. Helados (1 cucurucho)									

II. HUEVOS, CARNES, PESCADOS

Un plato o ración de 100-150 gr, excepto cuando se indique otra cosa	NUNCA O CASI NUNCA	AL MES	A LA SEMANA			AL DÍA			
		1 - 3	1	2 - 4	5 - 6	1	2 - 3	4 - 6	6 +
16. Huevos de gallina (uno)									
17. Pollo o pavo CON piel (1 ración o pieza)									
18. Pollo o pavo SIN piel (1 ración o pieza)									
19. Carne de ternera o vaca (1 ración)									
20. Carne de cerdo (1 ración)									
21. Carne de cordero (1 ración)									
22. Conejo o liebre (1 ración)									
23. Hígado (ternera, cerdo, pollo) (1 ración)									
24. Otras vísceras (sesos, corazón, mollejas) (1 ración)									
25. Jamón serrano o paletilla (1 loncha, 30 gr.)									
26. Jamón York, jamón cocido (1 loncha, 30 gr.)									
27. Carnes procesadas (salchichón, chorizo, morcilla, mortadela, salchichas, butifarra, sobrasada, 50 gr.)									
28. Patés, foie-gras (25 gr.)									
29. Hamburguesa (una, 50 gr.), albóndigas (3 unidades)									
30. Tocino, bacon, panceta (50 gr.)									
31. Pescado blanco: mero, lenguado, besugo, merluza, pescadilla,... (1 plato, pieza o ración)									
32. Pescado azul: sardinas, atún, bonito, caballa, salmón (1 plato, pieza o ración 130 gr.)									
33. Pescados salados: bacalao, salazones (1 ración, 60 gr. en seco)									
34. Ostras, almejas, mejillones y similares (6 unidades)									
35. Calamares, pulpo, chipirones, jibia (sepia) (1 ración, 200 gr.)									
36. Crustáceos: gambas, langostinos, cigalas, etc. (4-5 piezas, 200 gr.)									
37. Pescados y mariscos enlatados al natural (sardinas, anchoas, bonito, atún) (1 lata pequeña o media lata normal, 50 gr.)									
38. Pescados y mariscos en aceite (sardinas, anchoas, bonito, atún) (1 lata pequeña o media lata normal, 50 gr.)									

SUMCO 12637-03-1

Por favor, marque una única opción para cada alimento.

III. VERDURAS Y HORTALIZAS

Un plato o ración de 200 grs, excepto cuando se indique	CONSUMO MEDIO DURANTE EL AÑO PASADO								
	NUNCA O CASI NUNCA	AL MES	A LA SEMANA			AL DÍA			
		1 - 3	1	2 - 4	5 - 6	1	2 - 3	4 - 6	6 +
39. Acelgas, espinacas									
40. Col, coliflor, brócoles									
41. Lechuga, endivias, escarola (100 gr.)									
42. Tomate crudo (1, 150 gr)									
43. Zanahoria, calabaza (100 gr.)									
44. Judías verdes									
45. Berenjenas, calabacines, pepinos									
46. Pimientos (150 gr.)									
47. Espárragos									
48. Gazpacho andaluz (1 vaso, 200 gr.)									
49. Otras verduras (alcachofa, puerro, cardo, apio)									
50. Cebolla (media unidad, 50 gr.)									
51. Ajo (1 diente)									
52. Perejil, tomillo, laurel, orégano, etc. (una pizca)									
53. Patatas fritas comerciales (1 bolsa, 50 gr.)									
54. Patatas fritas caseras (1 ración, 150 gr.)									
55. Patatas asadas o cocidas									
56. Setas, níscalos, champiñones									

IV. FRUTAS

Una pieza o ración	CONSUMO MEDIO DURANTE EL AÑO PASADO								
	NUNCA O CASI NUNCA	AL MES	A LA SEMANA			AL DÍA			
		1 - 3	1	2 - 4	5 - 6	1	2 - 3	4 - 6	6 +
57. Naranja (una), pomelo (una), o mandarinas (dos)									
58. Plátano (uno)									
59. Manzana o pera (una)									
60. Fresas/fresones (6 unidades, 1 plato postre)									
61. Cerezas, picotas, ciruelas (1 plato de postre)									
62. Melocotón, albaricoque, nectarina (una)									
63. Sandía (1 tajada, 200-250 gr.)									
64. Melón (1 tajada, 200-250 gr.)									
65. Kiwi (1 unidad, 100 gr.)									
66. Uvas (un racimo, 1 plato postre)									
67. Aceitunas (10 unidades)									
68. Frutas en almíbar o en su jugo (2 unidades)									
69. Dátiles, higos secos, uvas-pasas, ciruelas-pasas (150 gr.)									
70. Almendras, cacahuetes, avellanas, pistachos, piñones (30 gr.)									
71. Nueces (30 gr.)									

72. ¿Cuántos días a la semana toma fruta como postre?	0	1	2	3	4	5	6	7

V. LEGUMBRES Y CEREALES

Un plato o ración (150 gr.)	CONSUMO MEDIO DURANTE EL AÑO PASADO								
	NUNCA O CASI NUNCA	AL MES	A LA SEMANA			AL DÍA			
		1 - 3	1	2 - 4	5 - 6	1	2 - 3	4 - 6	6 +
73. Lentejas (1 plato, 150 gr. cocidas)									
74. Alubias (pintas, blancas o negras) (1 plato, 150 gr. cocidas)									
75. Garbanzos (1 plato, 150 gr. cocidos)									
76. Guisantes, habas (1 plato, 150 gr. cocidas)									
77. Pan blanco, pan de molde (3 rodajas, 75 gr.)									
78. Pan negro o integral (3 rodajas, 75 gr.)									
79. Cereales desayuno (30 gr.)									
80. Cereales integrales: muesli, copos avena, all-bran (30 gr.)									
81. Arroz blanco (60 gr. en crudo)									
82. Pasta: fideos, macarrones, espaguetis, otras (60 gr. en crudo)									
83. Pizza (1 ración, 200 gr.)									

NODO	CENTRO	MÉDICO	PACIENTE	VISITA
0 0	0 0	0 0	0 0	0 0
1 1	1 1	1 1	1 1	1 1
2 2	2 2	2 2	2 2	2 2
3 3	3 3	3 3	3 3	3 3
4 4	4 4	4 4	4 4	4 4
5 5	5 5	5 5	5 5	5 5
6 6	6 6	6 6	6 6	6 6
7 7	7 7	7 7	7 7	7 7
8 8	8 8	8 8	8 8	8 8
9 9	9 9	9 9	9 9	9 9

marque así

así no marque

Por favor, marque una única opción para cada alimento.

VI. ACEITES Y GRASAS

Una cucharada o porción individual. Para freír, untar, mojar en el pan, para aliñar, o para ensaladas, utiliza en total:	CONSUMO MEDIO DURANTE EL AÑO PASADO								
	NUNCA O CASI NUNCA	AL MES	A LA SEMANA			AL DÍA			
		1 - 3	1	2 - 4	5 - 6	1	2 - 3	4 - 6	6 +
**84. Aceite de oliva (una cucharada sopera)**									
**85. Aceite de oliva extra virgen (una cucharada sopera)**									
**86. Aceite de oliva de orujo (una cucharada sopera)**									
**87. Aceite de maíz (una cucharada sopera)**									
**88. Aceite de girasol (una cucharada sopera)**									
**89. Aceite de soja (una cucharada sopera)**									
**90. Mezcla de los anteriores (una cucharada sopera)**									
**91. Margarina (porción individual, 12 gr.)**									
**92. Mantequilla (porción individual, 12 gr.)**									
**93. Manteca de cerdo (10 gr.)**									

94. Marca de aceite de oliva que usa habitualmente: ....

0 1 2 3 4 5 6 7 8 9
0 1 2 3 4 5 6 7 8 9

No marque aquí

VII. BOLLERÍA Y PASTELERÍA

CONSUMO MEDIO DURANTE EL AÑO PASADO	NUNCA O CASI NUNCA	AL MES	A LA SEMANA			AL DÍA			
		1 - 3	1	2 - 4	5 - 6	1	2 - 3	4 - 6	6 +
**95. Galletas tipo María (4-6 unidades, 50 gr.)**									
**96. Galletas integrales o de fibra (4-6 unidades, 50 gr.)**									
**97. Galletas con chocolate (4 unidades, 50 gr.)**									
**98. Repostería y bizcochos hechos en casa (50 gr.)**									
**99. Croissant, ensaimada, pastas de té u otra bollería industrial comercial… (uno, 50 gr)**									
**100. Donuts (uno)**									
**101. Magdalenas (1-2 unidades)**									
**102. Pasteles (uno, 50 gr.)**									
**103. Churros, porras y similares (1 ración, 100 gr.)**									
**104. Chocolates y bombones (30 gr.)**									
**105. Cacao en polvo-cacaos solubles (1 cucharada de postre)**									
**106. Turrón (1/8 de barra, 40 gr.)**									
**107. Mantecados, mazapán (90 gr.)**									

VIII. MISCELÁNEA

CONSUMO MEDIO DURANTE EL AÑO PASADO	NUNCA O CASI NUNCA	AL MES	A LA SEMANA			AL DÍA			
		1 - 3	1	2 - 4	5 - 6	1	2 - 3	4 - 6	6 +
**108. Croquetas, buñuelos, empanadillas, precocinados (una)**									
**109. Sopas y cremas de sobre (1 plato)**									
**110. Mostaza (una cucharadita de postre)**									
**111. Mayonesa comercial (1 cucharada sopera = 20 gr.)**									
**112. Salsa de tomate frito, ketchup (1 cucharadita)**									
**113. Picante: tabasco, pimienta, pimentón (una pizca)**									
**114. Sal (una pizca)**									
**115. Mermeladas (1 cucharadita)**									
**116. Azúcar (1 cucharadita)**									
**117. Miel (1 cucharadita)**									
**118. Snacks distintos de patatas fritas: gusanitos, palomitas, maíz, etc. (1 bolsa, 50 gr.)**									
**119. Otros alimentos de frecuente consumo:**									
**119.1**									
**119.2**									
**119.3**									

SUMCO 12637-03-2

Por favor, marque una única opción para cada alimento.

IX. BEBIDAS	CONSUMO MEDIO DURANTE EL AÑO PASADO								
	NUNCA O CASI NUNCA	AL MES	A LA SEMANA			AL DÍA			
		1 - 3	1	2 - 4	5 - 6	1	2 - 3	4 - 6	6 +
120. Bebidas carbonatadas con azúcar: bebidas con cola, limonadas, tónicas, etc. (1 botellín, 200 cc)									
121. Bebidas carbonatadas bajas en calorías, bebidas light (1 botellín, 200 cc)									
122. Zumo de naranja natural (1 vaso, 200 cc)									
123. Zumos naturales de otras frutas (1 vaso, 200 cc)									
124. Zumos de frutas en botella o enlatados (200 cc)									
125. Café descafeinado (1 taza, 50 cc)									
126. Café (1 taza, 50 cc)									
127. Té (1 taza, 50 cc)									
128. Mosto (100 cc)									
129. Vaso de vino rosado (100 cc)									
130. Vaso de vino moscatel (50 cc)									
131. Vaso de vino tinto joven, del año (100 cc)									
132. Vaso de vino tinto añejo (100 cc)									
133. Vaso de vino blanco (100 cc)									
134. Vaso de cava (100 cc)									
135. Cerveza (1 jarra, 330 cc)									
136. Licores, anís o anisetes… (1 copa, 50 cc)									
137. Destilados: whisky, vodka, ginebra, coñac (1 copa, 50 cc)									

138. ¿A que edad empezó a beber alcohol (vino, cerveza o licores), incluyendo el que toma con las comidas con regularidad (más de siete "bebidas" a la semana)?

Edad (años)

0	1	2	3	4	5	6	7	8	9	Decena
0	1	2	3	4	5	6	7	8	9	Unidad

139. ¿Cuántos años ha bebido alcohol con regularidad (más de siete "bebidas" a la semana)?

Años

0	1	2	3	4	5	6	7	8	9	Decena
0	1	2	3	4	5	6	7	8	9	Unidad

119. Otros alimentos de frecuente consumo

119.1 (No marque aquí)

0	1	2	3	4	5	6	7	8	9
0	1	2	3	4	5	6	7	8	9

119.2 (No marque aquí)

0	1	2	3	4	5	6	7	8	9
0	1	2	3	4	5	6	7	8	9

119.3 (No marque aquí)

0	1	2	3	4	5	6	7	8	9
0	1	2	3	4	5	6	7	8	9

Si durante el año pasado tomó vitaminas y/o minerales (incluyendo calcio) o productos dietéticos especiales (salvado, aceite de onagra, leche con ácidos grasos omega-3, flavonoides, etc.), por favor indique la marca y la frecuencia con que los tomó:

Marcas de los suplementos de vitaminas o minerales o de los productos dietéticos	CONSUMO MEDIO DURANTE EL AÑO PASADO								
	NUNCA O CASI NUNCA	AL MES	A LA SEMANA			AL DÍA			
		1 - 3	1	2 - 4	5 - 6	1	2 - 3	4 - 6	6 +
140.									
140.1									
140.2									

140 (No marque aquí)

0	1	2	3	4	5	6	7	8	9
0	1	2	3	4	5	6	7	8	9

140.1 (No marque aquí)

0	1	2	3	4	5	6	7	8	9
0	1	2	3	4	5	6	7	8	9

140.2 (No marque aquí)

0	1	2	3	4	5	6	7	8	9
0	1	2	3	4	5	6	7	8	9

SUMCO 12637-03-(Ref. 2)

Muchas gracias por su colaboración

SKIN CANCER:

THE EXPOSOME PARADIGM

CUESTIONARIO PARA EL DERMATÓLOGO

- **Diagnóstico de cáncer de piel**

  1. Carcinoma basocelular (Sí/no)
  2. Carcinoma espinocelular (Sí/no)
  3. Queratosis actínicas (Sí/no)
  4. Melanoma (Sí/no)

- **Localización:**

  1. Cabeza y cuello
  2. Tronco
  3. Extremidades superiores
  4. Extremidades inferiores

- **Diagnósticos previos de cáncer de piel (Sí/no)**

- **Desarrollo de metástasis (Sí/no)**

- **¿Consume alguno de esto fármacos de forma habitual?**

  1. Estatinas (Sí/no)
  2. Omeprazol ((Sí/no)
  3. IECAs (Captopril, enalapril, ramipril) (Sí/no)
  4. Metformina (Sí/no)
  5. Ansiolíticos, antidepresivos o hipnóticos (Sí/no)
  6. Hidroclorotiazida (Sí/no)
  7. Hidroxiurea (Sí/no)
  8. Antioxidantes (Sí/no)
  9. Vitamina D (Sí/no)

- **Antecedentes familiares de cáncer de piel**

  1. Sí
  2. No
